# How autoreactive thymocytes differentiate into regulatory versus effector CD4^+^ T cells after avoiding clonal deletion

**DOI:** 10.1038/s41590-023-01469-2

**Published:** 2023-03-23

**Authors:** Xuguang Tai, Alyssa Indart, Mirelle Rojano, Jie Guo, Nicolai Apenes, Tejas Kadakia, Marco Craveiro, Amala Alag, Ruth Etzensperger, Mohamed Elsherif Badr, Flora Zhang, Zhongmei Zhang, Jie Mu, Terry Guinter, Assiatu Crossman, Larry Granger, Susan Sharrow, Xuyu Zhou, Alfred Singer

**Affiliations:** 1 Experimental Immunology Branch, National Cancer Institute, National Institutes of Health, Bethesda, MD USA; 2 Key Laboratory of Pathogenic Microbiology and Immunology, Institute of Microbiology, Chinese Academy of Sciences, Beijing, China

**Keywords:** Biological sciences, Regulatory T cells, Autoimmunity

## Abstract

Thymocytes bearing autoreactive T cell receptors (TCRs) are agonist-signaled by TCR/co-stimulatory molecules to either undergo clonal deletion or to differentiate into specialized regulatory T (T_reg_) or effector T (T_eff_) CD4^+^ cells. How these different fates are achieved during development remains poorly understood. We now document that deletion and differentiation are agonist-signaled at different times during thymic selection and that T_reg_ and T_eff_ cells both arise after clonal deletion as alternative lineage fates of agonist-signaled CD4^+^CD25^+^ precursors. Disruption of agonist signaling induces CD4^+^CD25^+^ precursors to initiate Foxp3 expression and become T_reg_ cells, whereas persistent agonist signaling induces CD4^+^CD25^+^ precursors to become IL-2^+^ T_eff_ cells. Notably, we discovered that transforming growth factor-β induces Foxp3 expression and promotes T_reg_ cell development by disrupting weaker agonist signals and that Foxp3 expression is not induced by IL-2 except under non-physiological in vivo conditions. Thus, TCR signaling disruption versus persistence is a general mechanism of lineage fate determination in the thymus that directs development of agonist-signaled autoreactive thymocytes.

## Abstract (web summary)

Singer and colleagues show that the developmental fate of autoreactive CD4^+^ thymocytes is determined by the timing and duration of agonist signaling. Early agonist signaling induces clonal deletion, whereas late agonist signaling induces differentiation into Foxp3^+^ T_reg_ cells or IL-2^+^ T_eff_ cells depending on whether TGF-β disrupts TCR signaling.

## Main

Antigen receptors expressed on developing T cells are screened in the thymus for usefulness and autoreactive potential during the process of thymic selection^1^. Thymocytes with autoreactive TCRs have high affinity for self-ligands that, together with CD28 co-stimulatory molecules, generate agonist signals^2^. However, thymocytes are initially signaled by TCR ligands present in the thymic cortex to undergo positive selection and to migrate toward the thymic medulla. It is when they reach the cortico-medullary junction and thymic medulla that autoreactive thymocytes encounter co-stimulatory ligands that, together with high-affinity TCR ligands, stimulate agonist signaling^3,4^. Agonist signaling then induces autoreactive thymocytes to either undergo clonal deletion or to differentiate into T_reg_ or T_eff_ CD4^+^ T cells^5–7^. Notably, all three developmental fates impact self-tolerance: clonal deletion removes potentially lethal autoreactive TCR specificities^8^; T_reg_ cells control activation of T_eff_ cells, which limits their pathogenicity^9^; and T_eff_ cells produce IL-2, which promotes terminal T_reg_ differentiation and survival^5,10^. Nonetheless, it is not well understood how autoreactive thymocytes are signaled to achieve different fates and whether these fates are signaled simultaneously or sequentially during thymic selection.

T_reg_ development has been intensively studied and these studies have revealed a complex set of molecular requirements and mechanisms for inducing *Foxp3* gene expression and T_reg_ cell differentiation^11,12^. Among the complexities and conflicts that exist, TCR–CD28 signaling seems to both promote and inhibit *Foxp3* gene expression^11^ and mature T_reg_ cells seem to arise from two different T_reg_ precursors either independently of one another^13–15^ or as a result of a possibly shared precursor–progeny relationship^12^. Similarly, thymic T_reg_ development has been shown to require transforming growth factor (TGF)-β^16,17^ but the role of TGF-β remains uncertain^18,19^. Compared to T_reg_ generation, far less is understood about the differentiation of agonist-signaled thymocytes into autoreactive T_eff_ cells and little is known regarding how T_reg_/T_eff_ lineage choices are made.

The present study was undertaken to understand how autoreactive thymocytes achieve their different developmental fates. We report that autoreactive cell fates depend on the timing and duration of agonist signaling, with agonist signaling of early stage thymocytes inducing clonal deletion and agonist signaling of late-stage thymocytes generating CD25^+^ precursors that differentiate into Foxp3^+^ T_reg_ cells or IL-2^+^ T_eff_ cells. Notably, T_reg_ and T_eff_ cells are alternative lineage fates of CD25^+^ precursors whose fate choices are modulated by TGF-β, which disrupts weaker agonist signals but not stronger agonist signals that persist undisrupted. This study substantially alters current understanding of T_reg_ and T_eff_ cell development and reveals how agonist-signaled thymocytes achieve different developmental fates.

## Results

### Timing of agonist signaling

**Fig. 1:**
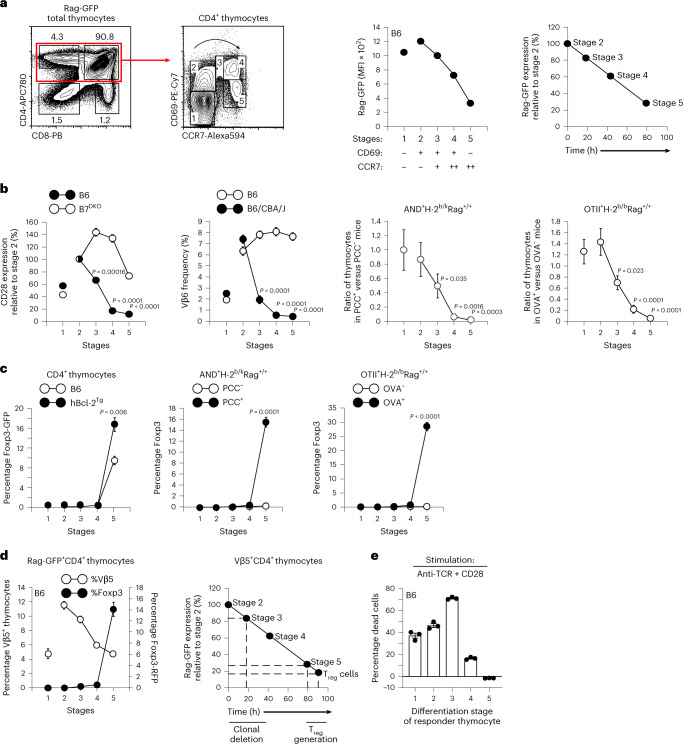
Timing of clonal deletion and T_reg_ generation in the thymus. **a**, Flow cytometric analysis of CD4^+^ thymocytes by CD69/CCR7 expression identifies five sequential stages of thymocyte differentiation (stages 1–5) in Rag-GFP.B6 mice as shown by steadily declining Rag-GFP expression (mean fluorescence intensity (MFI)) after stage 2. Differentiation time from stage 2 onward was calculated by the formula: time (h) = (100 − relative Rag-GFP MFI) / 0.9 based on a Rag-GFP half-life of 54–56 h and Rag-GFP content in stage 2 thymocytes set equal to 100. Data are representative of more than ten independent experiments. **b**, Surface CD28 expression on thymocytes from each strain was normalized relative to stage 2, which was set equal to 100%. Mean ± s.e.m. of five mice from three independent experiments. Clonal deletion induced by superantigens and conventional antigens. TCR-Vβ6 frequencies at each stage of differentiation in CD4^+^ thymocytes from indicated mice (*n* = 4, per strain). Relative number of TCR transgenic CD4^+^ thymocytes in antigen-positive compared to antigen-negative mice at each thymocyte stage. AND TCR^Tg^ (*n* = 4, per strain) and OT-II TCR^Tg^ (*n* = 11, per strain) were analyzed. Mean ± s.e.m. of three to six independent experiments. **c**, Frequency of Foxp3^+^ cells among CD4^+^ thymocytes at each thymocyte stage determined by Foxp3-GFP expression or intracellular Foxp3 staining. Mean ± s.e.m. of three independent experiments. **d**, Timing of TCR-Vβ5 deletion and T_reg_ generation in the same thymus. Frequency of TCR-Vβ5^+^ thymocytes and TCR-Vβ5^+^Foxp3^+^ cells among CD4^+^ thymocytes in Rag-GFP/Foxp3-RFP.B6 double-reporter mice (left). Differentiation time from stage 2 was calculated as in **a** (right). Mean ± s.e.m. of three experiments. **e**, TCR/CD28-signaled death of different stage thymocytes in overnight culture. Thymocytes at each stage of development were either stimulated with anti-TCR/CD28 or placed in medium cultures overnight. After overnight culture, dead cells were identified by ethidium bromide staining^2^ and frequency of dead cells in stimulation cultures was normalized to that in medium cultures at each thymocyte stage. Data are mean ± s.e.m. of triplicate cultures representative of three independent experiments; two-tailed unpaired Student’s *t-*test was used for **b**,**c**. Source data

**Extended Data Fig. 1:**
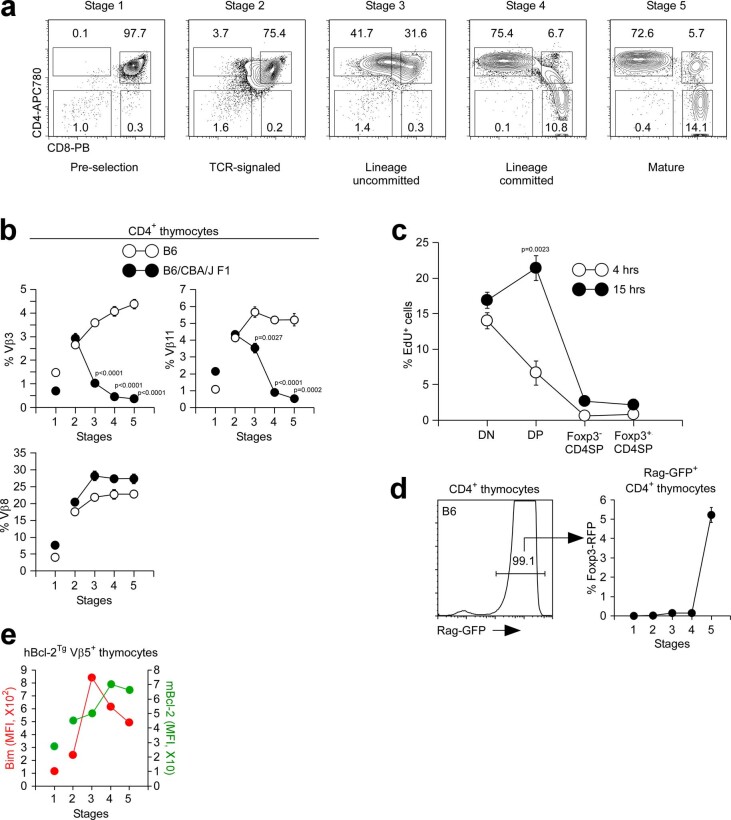
Timing of clonal deletion and Treg generation in the thymus. **a**, Analysis of CD4 and CD8 expression on thymocytes at sequential stages of thymocyte differentiation. Representative of 10 independent experiments. **b**, Frequencies of TCR-Vβ3^+^, -Vβ8^+^ and -Vβ11^+^ cells at each stage of CD4^+^ thymocyte differentiation. Mean ± SEM of 3 independent experiments. **c**, B6 mice were injected intra-peritoneally with EdU (1 mg/mouse) and then assessed after 4 h or 15 h for EdU labeling. Mean ± SEM of 3 mice for each time point, representative of two independent experiments. **d**, Frequency of Foxp3-RFP^+^ cells among different stage CD4^+^ thymocytes expressing the Rag-GFP^Tg^. Mean ± SEM of 5 independent experiments. **e**, Endogenous Bim and mBcl-2 protein expression in TCR-Vβ5^+^ cells among different stage CD4^+^ thymocytes from hBcl-2^Tg^ mice. Two-tailed unpaired t-test, mean ± SEM (**b** and **c**). Source data

The present study was undertaken to determine when and how autoreactive CD4^+^ thymocytes are agonist-signaled to pursue different developmental fates. Autoreactive thymocytes are initially signaled to undergo positive selection by TCR ligands in the thymic cortex, causing them to migrate toward the thymic medulla and to subsequently encounter co-stimulatory ligands that, together with high-affinity TCR ligands, induce agonist signaling. To track thymocyte development during thymic selection, we identified five sequential stages of thymic selection by differential surface expression of CD69 and CCR7 (Fig. 1a)^20^. Stage 1 cells are CD69^−^CCR7^−^ unsignaled pre-selection thymocytes, whereas cells at stages 2–5 are TCR-signaled thymocytes at progressively later stages of selection that express CD69 and/or CCR7 (Fig. 1a and Extended Data Fig. 1a). At stage 5, CCR7^+^ thymocytes lose surface CD69 expression and exit the thymus. We determined the time that it took for TCR-signaled CD4^+^ thymocytes to progress to stage 5 by using a *Rag2* promoter-driven transgene encoding green fluorescent protein (Rag-GFP). When *Rag* gene expression is silenced by TCR signaling, Rag-GFP synthesis ceases and intracellular GFP content declines with a linear half-life of 54–56 h (ref. ^21^), documenting that TCR-signaled stage 2 thymocytes sequentially differentiate into stage 5 thymocytes in ~80 h (Fig. 1a).

While clonal deletion and T_reg_ generation both require agonist signaling stimulated by TCR and CD28 co-stimulatory ligands^2,7,22–24^, it is not known whether these developmental fates are agonist-signaled simultaneously or sequentially during thymic selection. To address this issue, we first determined when thymocyte CD28 molecules are engaged by B7 co-stimulatory ligands (CD80 and CD86)^25^. By comparing CD28 on B6 and B7 double-knockout (B7^DKO^) thymocytes, we found that CD28-B7 interactions stimulated thymocytes to downregulate CD28 at stages 3 and 4, whereas later CD28 downregulation may be B7-independent (Fig. 1b).

We then assessed clonal deletion induced by the endogenous mammary tumor virus-9 (Mtv-9) superantigen to delete TCR-Vβ3, TCR-Vβ6 and TCR-Vβ11 thymocytes in I-E^+^ (B6xCBA/J) mice^26^. TCR-Vβ3, TCR-Vβ6 and TCR-Vβ11 frequencies were similar in stages 1 and 2 of deleting B6xCBA/J and non-deleting B6 thymocytes, but specifically declined in deleting strain thymocytes beginning at stage 3 (Fig. 1b and Extended Data Fig. 1b). We also assessed clonal deletion induced by conventional antigens in AND and OT-II TCR transgenic mice and observed that AND and OT-II thymocyte numbers specifically declined in antigen-positive mice beginning at stage 3 (Fig. 1b). Thus, clonal deletion is initiated at stage 3 when CD28-B7 engagements first occur.

While clonal deletion initiates at stage 3, Foxp3^+^ thymocytes did not appear until stage 5 (Fig. 1c). Notably, Foxp3^+^ cells did not arise earlier and undergo clonal deletion because early stage Foxp3^+^ cells would then appear among human Bcl-2 transgenic (hBcl-2^Tg^) thymocytes that are resistant to clonal deletion^7,27^ (Fig. 1c). Foxp3^+^ thymocytes also did not arise early and then proliferate to detectable numbers at stage 5 because Foxp3^+^ T_reg_ cells were unlabeled by in vivo 5-ethynyl-2′-deoxyuridine (EdU) injection, which labeled proliferating double-negative thymocytes and their double-positive progeny (Extended Data Fig. 1c). Finally, Foxp3^+^ cells were not stage 5 as a result of their recirculation back to the thymus from the periphery because Foxp3^+^ cells were still stage 5 among newly arising Rag-GFP^+^ thymocytes that had not exited the thymus^28,29^ (Extended Data Fig. 1d). We conclude that clonal deletion occurs before T_reg_ generation.

We determined the actual time after *Rag* gene termination that clonal deletion occurs and T_reg_ cells appear using double-reporter Rag-GFP/Foxp3-RFP mice whose TCR-Vβ5^+^ thymocytes are agonist-signaled in vivo by endogenous Mtv-9 superantigen associated with I-A^b^ molecules^30^. We found that partial deletion of TCR-Vβ5^+^ thymocytes occurred at stage 3 and that TCR-Vβ5^+^ T_reg_ cells appeared at stage 5 which, by Rag-GFP content, occurred at 20 h and 90 h, respectively, after *Rag* gene termination (Fig. 1d).

To understand why clonal deletion mainly affected early stage thymocytes, we stimulated thymocytes at each stage with immobilized anti-TCR/CD28 monoclonal antibodies and found that agonist signaling induced death of most stage 3 thymocytes but few stage 4–5 thymocytes (Fig. 1e). To understand why, we compared thymocyte expression of the pro-apoptotic protein Bim and the anti-apoptotic protein murine Bcl-2 (mBcl-2) (Extended Data Fig. 1e). Bim peaked at stage 3 and Bcl-2 peaked at stages 4–5 (Extended Data Fig. 1e), suggesting that Bim promoted death of agonist-signaled stage 3 thymocytes, whereas Bcl-2 prevented death of agonist-signaled thymocytes at stages 4–5.

### Late agonist signaling of T_reg_ development

**Fig. 2:**
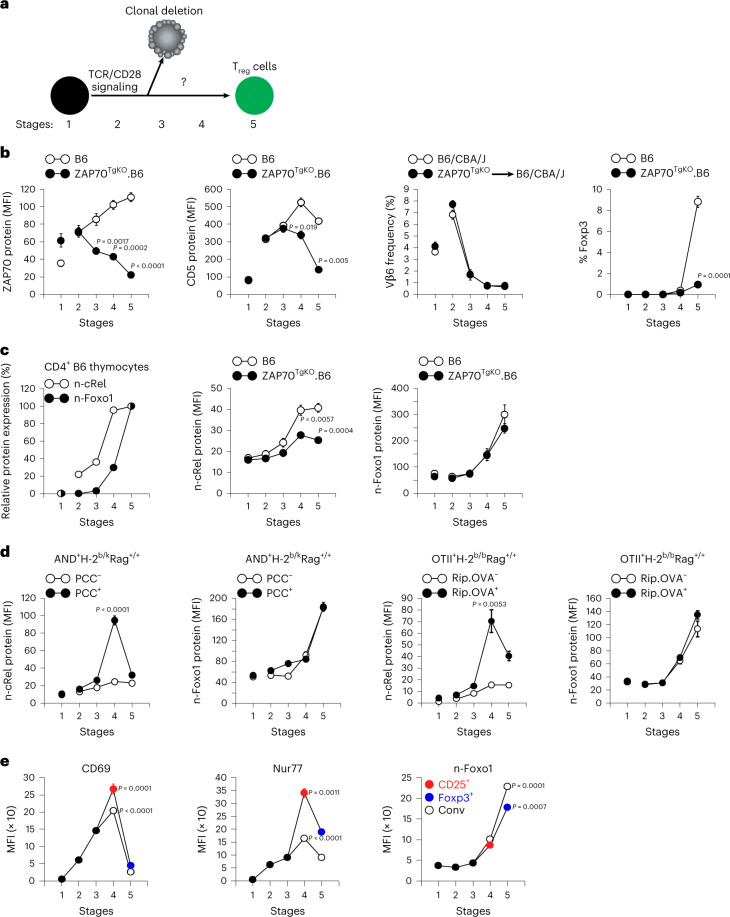
Assessment of clonal deletion and T_reg_ generation in the absence of late-stage agonist signaling. **a**, Schematic illustration that TCR/CD28-mediated agonist signaling of early stage thymocytes induces clonal deletion but it is not known whether it also induces T_reg_ cells. **b**, Diminished or absent TCR signal transduction in late-stage CD4^+^ thymocytes from ZAP70^TgKO^ mice and its effect on clonal deletion and T_reg_ generation. Different stage CD4^+^ thymocytes were assessed for expression of the indicated proteins (left) or frequencies of TCR-Vβ6 and Foxp3^+^ cells. TCR-Vβ6^+^ frequencies in CD4^+^ thymocytes from intact B6xCBA/J mice and CD4^+^ thymocytes of ZAP70^TgKO^ (CD45.1^−^) origin that developed in irradiated B6xCBA/J (CD45.1^+^) host mice. **c**, Effect of late-stage agonist signaling on n-cRel and n-Foxo1 upregulation. n-cRel and n-Foxo1 in different stage B6 thymocytes were expressed relative to stage 5, which was set equal to 100% (left). Intracellular staining for n-cRel and n-Foxo1 was compared in different stage CD4^+^ thymocytes from ZAP70^TgKO^ B6 (*n* = 3) and control B6 (*n* = 3) mice (middle and right). **d**, Impact of antigen-specific agonist signaling on n-cRel and n-Foxo1 expression in TCR transgenic thymocytes at different stages of differentiation. n-cRel and n-Foxo1 were quantified in different stage CD4^+^ thymocytes from AND and OT-II TCR transgenic mice expressing or lacking their agonist antigen (PCC or OVA). **e**, In vivo TCR signaling is disrupted at stage 5. Analysis of CD69, Nur77-GFP and n-Foxo1 expression in different stage CD4^+^ thymocytes. CD25^+^ stage 4 thymocytes, Foxp3^+^ stage 5 thymocytes and conventional (CD25^+^, Foxp3^+^ and conventional (conv)) thymocytes are indicated. Data were analyzed by two-tailed unpaired Student’s *t*-test for **b**–**e** and show mean ± s.e.m. of three experiments. Source data

**Extended Data Fig. 2:**
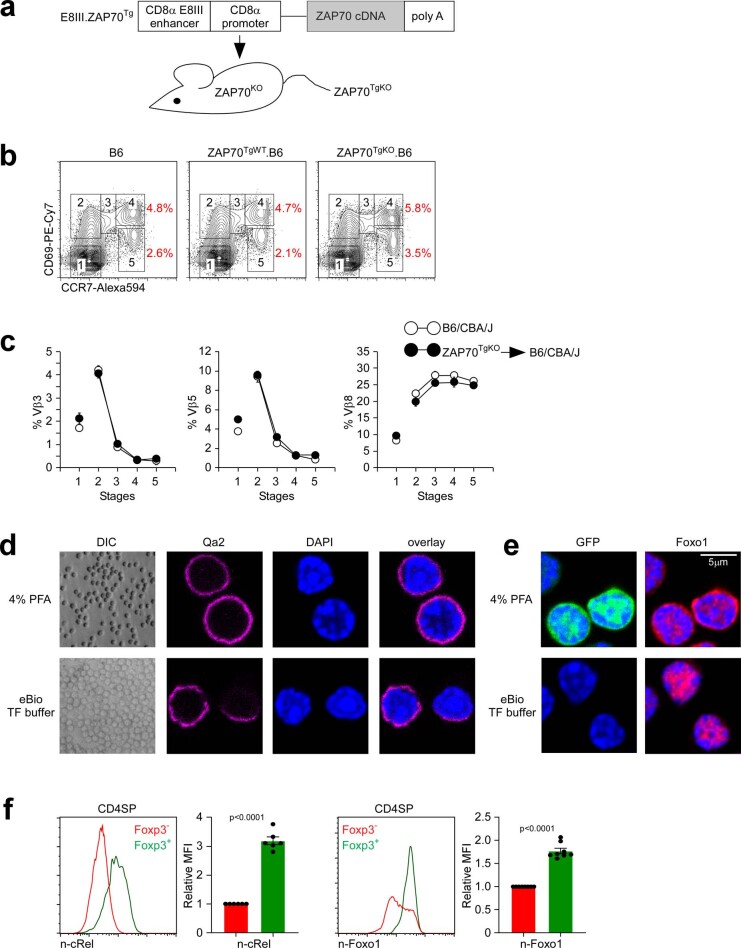
Assessment of clonal deletion and Treg generation in the absence of late-stage agonist signaling. **a**, A schematic representation of the ZAP70^TgKO^ mouse line. Mouse ZAP70 cDNA was put under the control of the *Cd8*-E8_III_ promoter/enhancer to generate the ZAP70^Tg^ which was then introduced into ZAP70^KO^ mice to generate ZAP70^TgKO^ mice. **b**, Analysis of CD4^+^ thymocytes from the indicated mice. **c**, TCR-Vβ3^+^, -Vβ5^+^ and -Vβ8^+^ frequencies in CD4^+^ thymocytes from intact B6xCBA/J mice and CD4^+^ thymocytes of ZAP70^TgKO^ (CD45.1^−^) origin that developed in irradiated B6xCBA/J (CD45.1^+^) host mice. **d**, Electronically purified Foxp3-GFP^+^ Tregs were first surface stained for Qa2 and then fixed with either 4% PFA or eBioscience Transcription Factor Staining Buffer (eBio TF buffer). The hypotonicity of eBio TF buffer was revealed by differential interference contrast (*DIC*) images and impaired cytoplasmic membrane integrity. **e**, Sorted Foxp3-GFP^+^ Tregs were fixed with either 4% PFA or eBio TF buffer and then the intracellular distribution of GFP and Foxo1 were detected. Note the loss of total GFP and cytosolic Foxo1 after eBio TF buffer treatment. Depicted representative of 3 independent experiments (**d** and **e**). **f**, Relative n-cRel and n-Foxo1 expression in Foxp3^+^ Tregs. The n-cRel and n-Foxo1 content in Foxp3^-^ CD4SP were set as 1. Two-tailed unpaired t-test, mean ± SEM of 5 independent experiments. Source data

Even though late-stage 4–5 thymocytes survive agonist signaling, it is not known whether agonist signaling of late-stage thymocytes induces their differentiation into T_reg_ cells (Fig. 2a). To answer this question, we constructed ZAP70^TgKO^ mice whose thymocytes lack late agonist signals. ZAP70^TgKO^ mice consist of Zap70^KO^ host mice with a ZAP70 transgene driven by the *Cd8*-E8_III_ promoter/enhancer element that only induces ZAP70 expression in pre-selection double-positive thymocytes and is then silenced^31,32^ (Extended Data Fig. 2a). Consequently, ZAP-70 protein expression declines throughout thymic selection, impairing CD5 upregulation on stage 4–5 thymocytes but not other aspects of positive selection (Fig. 2b and Extended Data Fig. 2b). Of note, Foxp3^+^ T_reg_ generation was abrogated in ZAP70^TgKO^ thymocytes, whereas TCR-Vβ-specific clonal deletion was unaffected (Fig. 2b and Extended Data Fig. 2c). Thus, early agonist signaling induces clonal deletion whereas T_reg_ generation requires late agonist signaling. We conclude that agonist signaling at different stages of selection induces different fates.

### Consequences of agonist signaling and its disruption

To understand how late agonist signaling generates T_reg_ cells, we assessed thymocyte expression of the transcription factors cRel and Foxo1 that promote *Foxp3* gene expression^33–36^. We quantified cRel and Foxo1 specifically in the cell nucleus by pretreating thymocytes with a hypotonic fixation buffer that retains only DNA-bound proteins (Extended Data Fig. 2d,e). In this way, we found that nuclear cRel (n-cRel) and nuclear Foxo1 (n-Foxo1) are both upregulated in Foxp3^+^ thymocytes relative to conventional (Foxp3^−^) thymocytes (Extended Data Fig. 2f), but they are upregulated at different stages. n-cRel is upregulated at stage 4 and n-Foxo1 is upregulated at stage 5; and n-cRel upregulation is impaired in ZAP70^TgKO^ thymocytes while n-Foxo1 upregulation is unimpaired (Fig. 2c). Additionally, TCR transgenic thymocytes revealed that n-cRel upregulation is enhanced by antigen-induced agonist signaling, whereas n-Foxo1 upregulation is unaffected (Fig. 2d). Thus, late agonist signaling enhances n-cRel upregulation but not n-Foxo1 upregulation.

To identify what upregulates n-Foxo1, we examined TCR signaling intensity as reported by CD69 and Nurr77 expression^37^. In conventional Foxp3^−^ thymocytes, TCR signaling increased until stage 4 and was then disrupted at stage 5 when n-Foxo1 was upregulated (Fig. 2e, white circles). Because CD25^+^ thymocytes are thought to be T_reg_ precursors^13^, we assessed developing T_reg_ cells by examining stage 4 thymocytes that were CD25^+^ and stage 5 thymocytes that were Foxp3^+^ (Fig. 2e, colored circles). We found that CD25^+^ stage 4 thymocytes were strongly TCR-signaled and that TCR signaling was disrupted in Foxp3^+^ stage 5 thymocytes when n-Foxo1 was upregulated (Fig. 2e, colored circles). Because signaling disruption and n-Foxo1 upregulation both occurred at stage 5, we thought that signaling disruption might induce n-Foxo1 upregulation.

**Extended Data Fig. 3:**
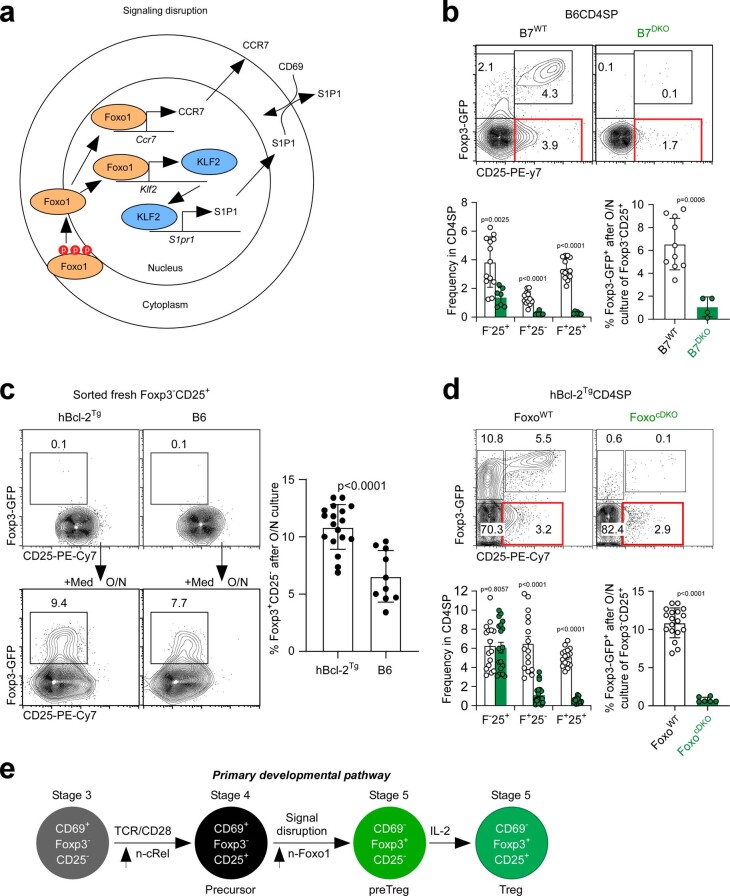
Signaling disruption and Treg generation. **a**, Regulation of n-Foxo1 expression and its target gene expression. **b**, CD4SP thymocytes from the indicated strains were analyzed by flow cytometry (top and lower left panels, n = 13 for B7^WT^ and n = 7 for B7^DKO^). Foxp3^-^CD25^+^ Treg precursors from the indicated strains were electronically sorted and placed in O/N medium cultures, after which they were assessed for Foxp3 expression (lower right panel). Dots represent individual mice, n = 10 for B7^WT^ and n = 4 for B7^DKO^. **c**, Foxp3^-^CD25^+^ CD4SP thymocytes from hBcl-2^Tg^ (n = 18) or B6 (n = 10) mice were placed in O/N medium cultures and then analyzed. Dots represent individual mice assayed in 9 independent experiments. **d**, CD4SP thymocytes from the indicated strains were analyzed as in **b**. For fresh cells (top and lower left panels), n = 16 for Foxo^WT^ and n = 19 for Foxo^cDKO^. For cultured cells (lower right panel), n = 18 for Foxo^WT^ and n = 6 for Foxo^cDKO^. **e**, Schematic of the primary Treg developmental pathway. Agonist signaling disruption occurs in Stage 4 CD25^+^ precursor thymocytes during their differentiation into stage 5 cells, which upregulates n-Foxo1 expression and induces Foxp3 gene expression. Two-tailed unpaired t-test, mean ± SEM (**b**, **c** and **d**).

Indeed, there is a known molecular mechanism by which TCR signaling disruption upregulates n-Foxo1 expression (Extended Data Fig. 3a)^38^. Disruption of TCR signaling causes dephosphorylation of Foxo1 proteins which translocate from the cytosol to the nucleus, increasing n-Foxo1 expression. Because n-Foxo1 induces transcription of its target genes, signaling disruption increases surface CCR7 expression and decreases surface CD69 expression^39–41^ (Extended Data Fig. 3a). Thus, in this perspective, disruption of late agonist signaling is predicted to have the following molecular consequences: upregulation of n-Foxo1, increased CCR7 and decreased CD69.

**Fig. 3:**
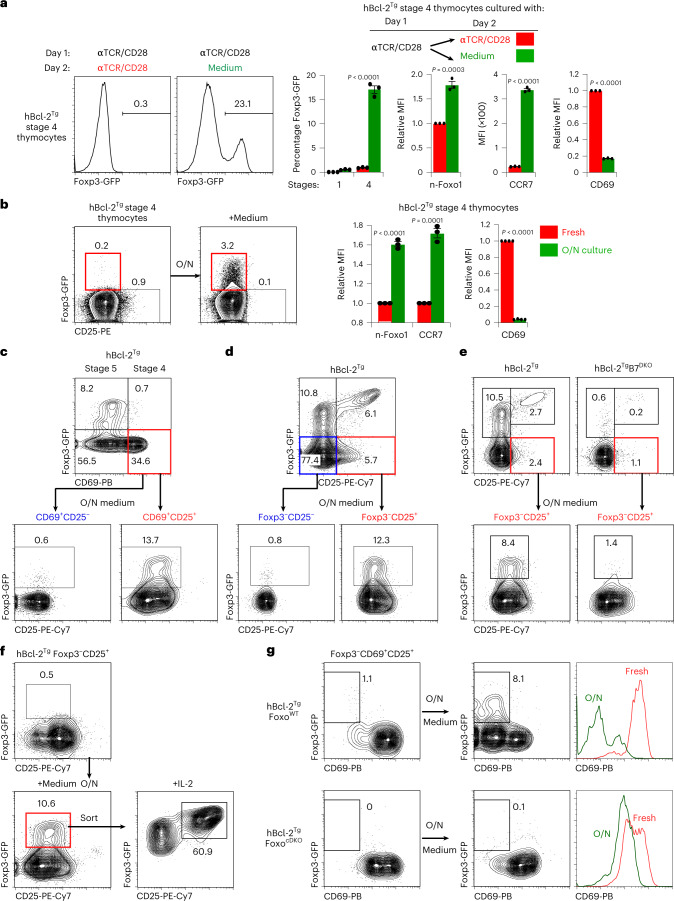
Generation of Foxp3^+^ T_reg_ cells in vitro. **a**, In vitro signaled stage 4 thymocytes express Foxp3 only after placement in medium culture. Electronically sorted stage 1 and stage 4 CD4^+^ thymocytes from hBcl-2^Tg^ mice were cultured in vitro for 24 h with immobilized anti-TCR/CD28 monoclonal antibodies for 1 day and then either continued or transferred into medium cultures for day 2, after which cultured thymocytes were analyzed. Data show mean ± s.e.m. of triplicate cultures representative of four independent experiments. **b**, In vivo signaled stage 4 thymocytes express Foxp3 after medium culture. Sorted stage 4 CD4^+^ thymocytes from hBcl-2^Tg^ mice were placed in O/N medium cultures and then assessed for expression of the indicated proteins. Data show mean ± s.e.m. of triplicate cultures representative of five independent experiments. **c**, Foxp3 precursors are Foxp3^−^CD69^+^CD25^+^ thymocytes. CD4SP thymocytes from hBcl-2^Tg^ mice were electronically sorted as indicated, placed in O/N medium cultures and then analyzed. Data are representative of six independent experiments. **d**, CD4SP thymocytes from hBcl-2Tg mice were electronically sorted as indicated, placed in O/N medium cultures and then analyzed. Data are representative of nine independent experiments. **e**, CD4SP thymocytes from the indicated strains were analyzed by flow cytometry. Where indicated, Foxp3^−^CD25^+^ thymocytes were electronically sorted and placed in O/N medium cultures, after which they were assessed for Foxp3 and CD25 expression. Results for each strain are representative of five independent experiments. **f**, Sorted Foxp3^−^CD25^+^ thymocytes from hBcl-2Tg mice were placed in O/N medium cultures; cells that became Foxp3-GFP^+^ were then purified again by electronic sorting, placed in O/N cultures containing IL-2 and assessed for Foxp3-GFP and CD25 expression. Data are representative of three independent experiments. **g**, Purified Foxp3^−^CD69^+^CD25^+^ thymocytes from the indicated mice were placed in O/N medium cultures and then analyzed. Data are representative of six independent experiments. Numbers in flow cytometry plots indicate percentages. Data were analyzed by two-tailed unpaired Student’s *t*-test (**a**,**b**).

To test whether Foxp3 gene induction requires agonist signaling to be disrupted, we stimulated thymocytes in vitro with immobilized anti-TCR/CD28 monoclonal antibodies (hBcl-2^Tg^ thymocytes were used to minimize thymocyte death in these stimulation cultures^2,27^; Fig. 3a). Notably, continuous agonist signaling did not generate Foxp3^+^ cells, but signaling disruption by transfer into medium cultures on day 2 did generate Foxp3^+^ cells (Fig. 3a). Additionally, signaling disruption upregulated n-Foxo1, increased CCR7 and decreased CD69 as predicted (Fig. 3a). Thus, Foxp3 gene induction requires agonist signaling disruption.

Because autoreactive thymocytes are agonist-signaled by in vivo self-ligands, simply placing stage 4 thymocytes into medium culture should cause the small amount of thymocytes that were agonist-signaled in vivo to become Foxp3^+^ in vitro (Fig. 3b). In fact, we found that placing stage 4 (CD69^+^) thymocytes into medium cultures generated Foxp3^+^ cells and resulted in upregulated n-Foxo1, increased CCR7 and decreased CD69 (Fig. 3b). To better understand the in vitro generation of Foxp3^+^ cells, we wished to identify the stage 4 thymocyte subset that became Foxp3^+^ in vitro (Fig. 3c). To do so, we sorted stage 4 (CD69^+^) thymocytes into O/N medium cultures and found that Foxp3^+^ cells arose predominantly from the CD25^+^ subset (Fig. 3c,d). To determine whether CD25^+^ precursors were agonist-signaled in vivo, we compared in vitro differentiation of CD25^+^ thymocytes from wild-type (WT) and co-stimulation-deficient B7^DKO^ mice. In fact, B7^DKO^CD25^+^ thymocytes were unable to become Foxp3^+^ in vitro (Fig. 3e and Extended Data Fig. 3b). Note that we utilized hBcl-2^Tg^ thymocytes to minimize in vitro thymocyte death and that results with B6 thymocytes were similar though cell recoveries were lower (Extended Data Fig. 3c).

Notably, we observed that in vitro-generated Foxp3^+^ cells resembled in vivo Foxp3^+^CD25^−/lo^ thymocytes that were named ‘preT_reg_’ cells because they differentiated into Foxp3^+^CD25^+^ mature T_reg_ cells when IL-2 signaled^14^. In fact in vitro-generated Foxp3^+^ cells also became Foxp3^+^CD25^+^ mature T_reg_ cells when they were IL-2 signaled (Fig. 3f), indicating that in vitro-generated Foxp3^+^ cells are phenotypically and functionally preT_reg_ cells.

Finally, we assessed whether in vitro generation of Foxp3^+^ preT_reg_ cells was Foxo-dependent. To do so, we examined in vitro differentiation of CD25^+^ precursors from Foxo-deficient mice^34,35^ (Fig. 3g and Extended Data Fig. 3d). In fact CD25^+^ thymocytes from Foxo1/o3 conditional double-knockout (Foxo^cDKO^) mice failed to differentiate into Foxp3^+^ cells and had impaired CD69 downregulation (Fig. 3g and Extended Data Fig. 3d), documenting that the molecular consequences of in vitro signaling disruption and Foxp3^+^ cell generation are Foxo-dependent. Together these in vitro findings identify the ‘primary T_reg_ developmental pathway’ as consisting of agonist-signaled CD25^+^ precursors that differentiate into Foxp3^+^CD25^−^ preT_reg_ cells and then become Foxp3^+^CD25^+^ mature T_reg_ cells (schematized in Extended Data Fig. 3e).

### Primary T_reg_ developmental pathway in vivo

**Fig. 4:**
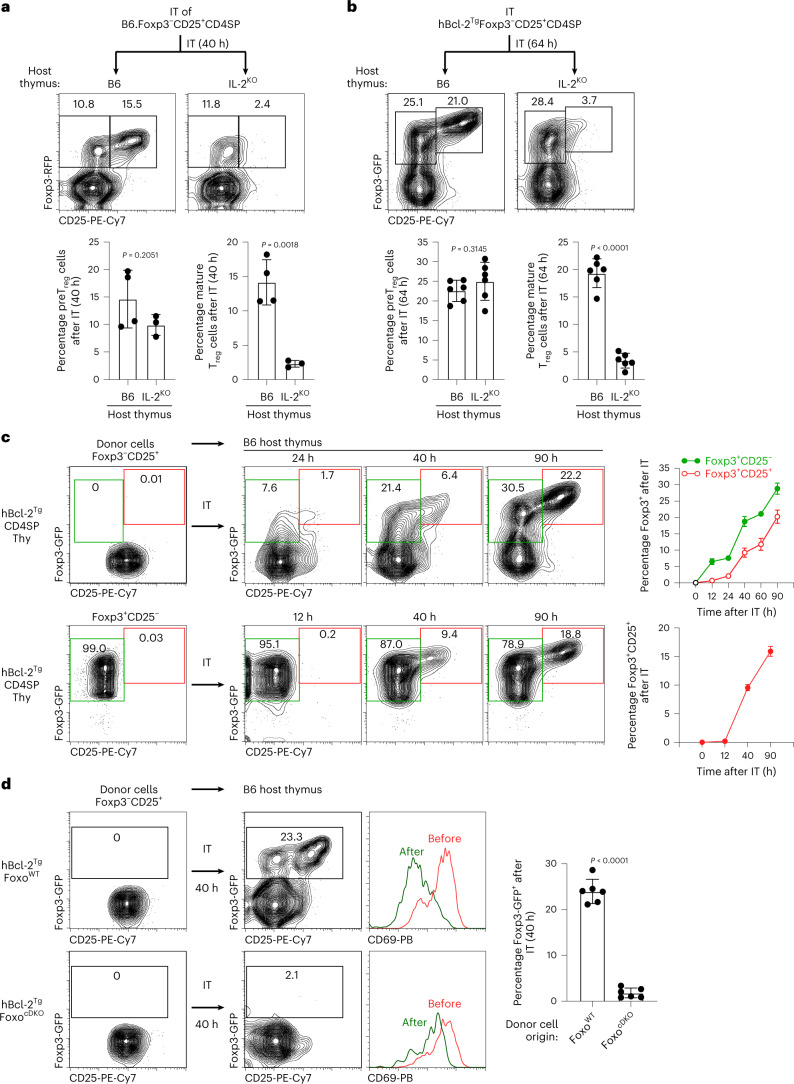
In vivo generation of Foxp3^+^ T_reg_ cells after intra-thymic transfer. **a**,**b**, Purified Foxp3^−^CD25^+^ CD4 SP thymocytes (CD45.2^+^) from B6 or hBcl-2^Tg^ mice were injected into the thymus of CD45.1^+^ B6 (*n* = 4 or 6 for B6 or hBcl-2^Tg^ donor cells) or IL-2^KO^ (*n* = 3 or 6 for B6 or hBcl-2^Tg^ donor cells) congenic hosts. At the indicated times after injection, thymi were recovered and phenotype of donor populations was determined. Dots represent individual mice assayed in three independent experiments. IT, intra-thymic transfer. **c**, Purified Foxp3^−^CD25^+^ or Foxp3^+^CD25^−^ CD4 SP thymocytes from hBcl-2^Tg^ mice (CD45.2^+^) were injected into the thymus of CD45.1^+^ B6 congenic hosts. At the indicated time of post injection, thymi were recovered and phenotype of donor populations was determined. Two to five host mice per each time point were analyzed in two to three independent experiments (*n* = 4 (12 h), *n* = 5 (24 h), *n* = 4 (40 h), *n* = 2 (60 h) and *n* = 2 (90 h) for Foxp3^−^CD25^+^ thymocytes, *n* = 1 (12 h), *n* = 4 (40 h) and *n* = 5 (90 h) for Foxp3^+^CD25^−^ thymocytes). **d**, Purified Foxp3^−^CD25^+^ CD4 SP thymocytes from the indicated mice were injected into the thymus of CD45.1^+^ B6 congenic hosts. After 40 h, thymi were recovered and phenotype of donor populations was determined. Dots represent individual mice assayed in three independent experiments. Numbers in flow cytometry plots indicate percentages. Data were analyzed by two-tailed unpaired Student’s *t*-test and show mean ± s.e.m. (**a**,**b**,**d**). Source data

**Extended Data Fig. 4:**
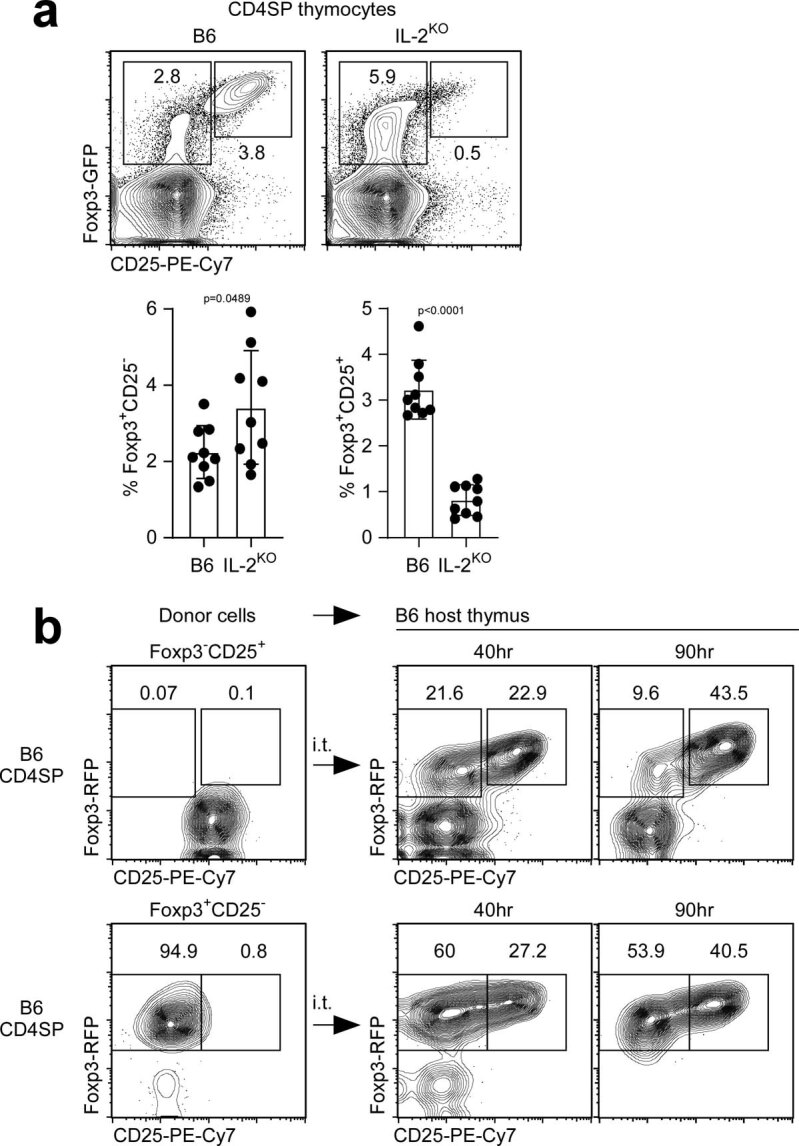
Verification of primary Treg developmental pathway in vivo. **a**, CD4SP thymocytes from the indicated strains were analyzed by flow cytometry (top panels). Depicted are frequencies of Foxp3^+^CD25^-^ preTregs and Foxp3^+^CD25^+^ mature Tregs (lower panels). Dots represent individual mice (n = 9 for both strains) assayed in 5 independent experiments. **b**, Foxp3^−^CD25^+^ or Foxp3^+^CD25^−^ CD4SP thymocytes from Foxp3RFP reporter positive B6 (CD45.2^+^) mice were injected into B6 (CD45.1^+^) host thymus. Donor T cells were analyzed after 40 hr or 90 hr. Representative of 4 experiments. Two-tailed unpaired t-test, mean ± SEM (**a**).

We then asked whether Foxp3^+^ T_reg_ cells arise in vivo via the primary developmental pathway. We performed intra-thymic transfer experiments with WT CD25^+^ precursors from B6 or hBcl-2^Tg^ mice and observed that they differentiated in host thymi into Foxp3^+^ thymocytes (Fig. 4a,b). Notably, their differentiation into Foxp3^+^CD25^−^ preT_reg_ cells was IL-2-independent, whereas their differentiation into Foxp3^+^CD25^+^ mature T_reg_ cells required IL-2 (Fig. 4a,b and Extended Data Fig. 4a). Time-course assessment revealed that CD25^+^ precursors differentiated first into Foxp3^+^CD25^−^ preT_reg_ cells (Fig. 4c) and that preT_reg_ cells then differentiated into Foxp3^+^CD25^+^ mature T_reg_ cells as reported^42,43^ (Fig. 4c), with hBcl-2^Tg^ and B6 donor thymocytes generating similar results (Fig. 4c and Extended Data Fig. 4b).

Having documented that in vivo Foxp3^+^ preT_reg_ generation was IL-2-independent, we examined its Foxo-dependence (Fig. 4d). Indeed, intra-thymic transfer into B6 host thymi revealed that Foxo-deficient CD25^+^ precursors could not differentiate into Foxp3^+^ thymocytes and had impaired CD69 downregulation (Fig. 4d), documenting that the molecular consequences of in vivo signaling disruption and Foxp3^+^ preT_reg_ generation were strictly Foxo-dependent as per the primary T_reg_ developmental pathway (Extended Data Fig. 3e).

### TGF-β disrupts late agonist signaling for Foxp3 expression

Because in vivo generation of Foxp3^+^ preT_reg_ cells requires agonist signaling disruption to upregulate n-Foxo1, we thought an in vivo mechanism must exist to disrupt agonist signaling. We further wondered if that mechanism involved TGF-β because the role of TGF-β in T_reg_ generation is incompletely understood^16–19,44^.

**Fig. 5:**
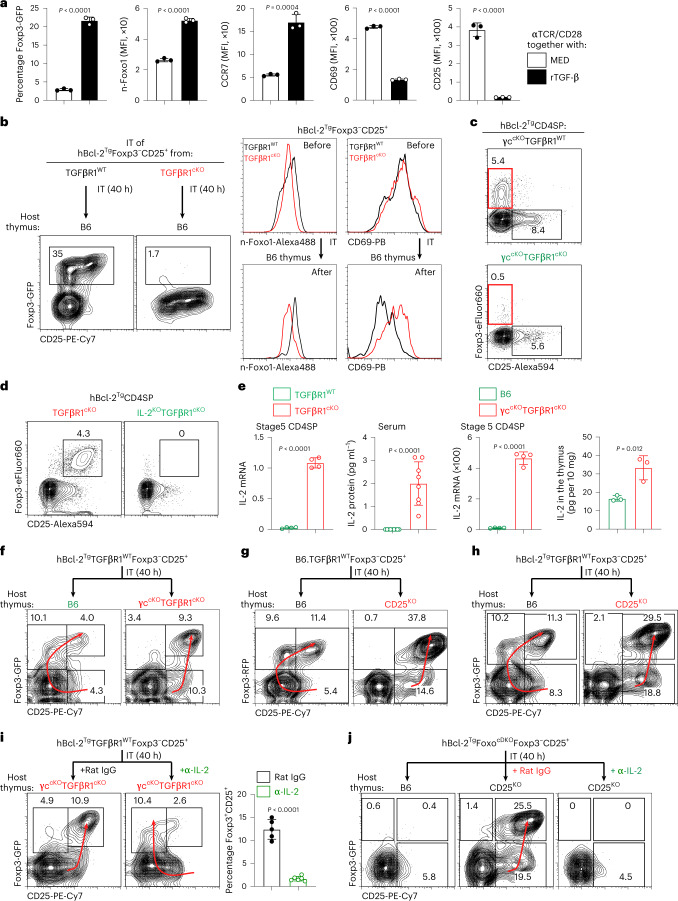
TGF-β and T_reg_ development. **a**, TGF-β disrupts in vitro agonist signaling to induce Foxp3^+^ thymocytes. Conventional (Foxp3^−^CD25^−^) CD4SP thymocytes from B6 mice were stimulated in vitro with immobilized anti-TCR/CD28 monoclonal antibodies alone or together with rTGF-β for 2 d, after which cultured thymocytes were analyzed for the expression of indicated proteins. Mean ± s.e.m. of triplicate cultures representative of five independent experiments. **b**, Foxp3^−^CD25^+^ CD4SP thymocytes from the indicated mice were injected into the thymus of CD45.1^+^ B6 hosts. After 40 h, phenotype of donor populations was determined. Representative of three independent experiments. **c**,**d**, CD4SP thymocytes from the indicated strains were analyzed by flow cytometry. Representative of four to six independent experiments. **e**, IL-2 mRNA expression in stage 5 CD4SP thymocytes or IL-2 protein in the serum or in the thymus of indicated mice. Data are mean ± s.e.m. of four technical replicates representative of four independent experiments for IL-2 mRNA and mean ± s.e.m. for IL-2 protein. Dots represent individual mice for protein analysis. **f**, Foxp3^−^CD25^+^ CD4SP thymocytes from hBcl-2^Tg^ mice (CD45.1^+^) were injected into the thymus of CD45.2^+^ B6 (*n* = 7) or γc^cKO^TGFβR1^cKO^ (*n* = 4) hosts. After 40 h, phenotype of donor populations was determined. Representative of three independent experiments. **g**,**h**, Foxp3^−^CD25^+^ CD4SP thymocytes from B6 or hBcl-2^Tg^ mice (CD45.1^+^) were injected into the thymus of CD45.2^+^ B6 or CD25^KO^ hosts. After 40 h, phenotype and frequency analysis of the donor populations were determined. **i**, Foxp3^−^CD25^+^ CD4SP thymocytes from hBcl-2^Tg^ mice (CD45.1^+^) were injected into the thymus of CD45.2^+^ γc^cKO^TGFβR1^cKO^ hosts that had received either rat IgG or anti-IL-2-neutralizing antibodies. After 40 h, the phenotype of donor populations was determined. Dots represent individual mice assayed in three independent experiments. **j**, Foxp3^−^CD25^+^ CD4SP thymocytes from hBcl-2^Tg^Foxo^cDKO^ mice (CD45.1^+^) were injected into the thymus of CD45.2^+^ B6 or CD25^KO^ hosts that had received either rat IgG or anti-IL-2-neutralizing antibodies. After 40 h, phenotype of donor populations was determined. Red arrows in **f**–**j** indicate the primary (left) and alternative (right) developmental pathways. Numbers in the flow cytometry plots indicate percentages. Data were analyzed by two-tailed unpaired Student’s *t*-test and show mean ± s.e.m. (**a**,**e**,**i**).

To determine whether TGF-β disrupts agonist signaling, we added recombinant TGF-β (rTGF-β) to anti-TCR/CD28 in vitro-stimulation cultures (Fig. 5a). Notably, despite continuous anti-TCR/CD28 engagement, addition of rTGF-β induced Foxp3^+^ cells, upregulated n-Foxo1, increased CCR7 and decreased CD69 all of which are molecular indicators of signaling disruption (Fig. 5a). Additionally, we found that CD25 expression, which was upregulated by agonist signaling, was also inhibited by rTGF-β (Fig. 5a). Together, these results strongly indicate that TGF-β disrupts agonist signaling whose mechanism was previously suggested^45,46^.

**Extended Data Fig. 5:**
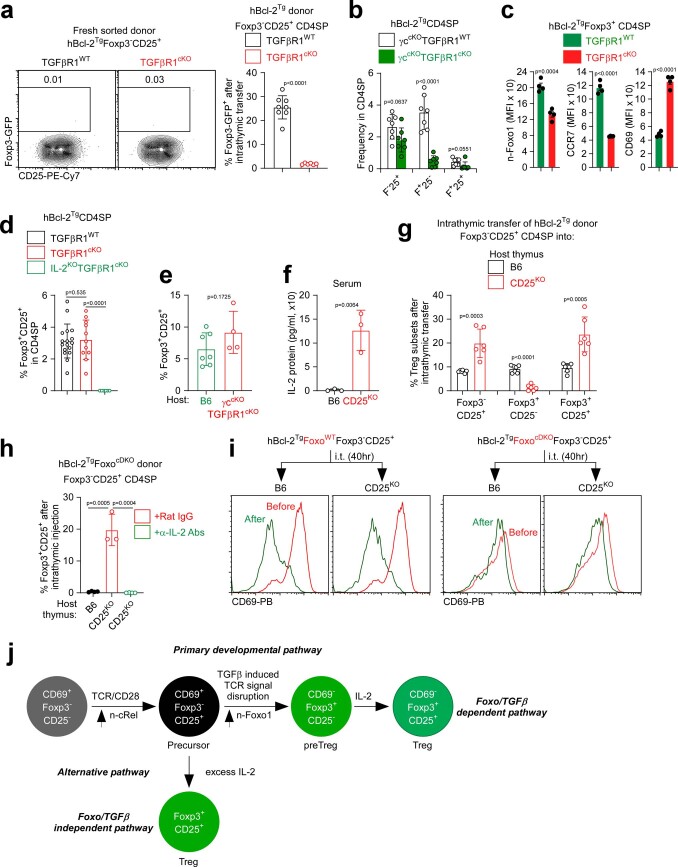
TGFβ signaling and Treg generation via the alternative pathway. **a**, Foxp3^−^CD25^+^ donor thymocytes (left) were injected into the thymus of B6 congenic hosts. Depicted are frequencies of Foxp3-GFP^+^ Tregs developed from donor cells (right). N = 8 for TGFβR1^WT^ donor cells and n = 6 for TGFβR1^cKO^ donor cells, assayed in 3 independent experiments. **b**, Analysis of CD4SP from the indicated strains (n = 7 per strain, assayed in 4 independent experiments). **c**, Quantification of n-Foxo1, surface CCR7 and CD69 on Foxp3^+^ CD4SP (n = 4 per strain). Mean ± SEM, 2 independent experiments. **d**, Frequencies of Foxp3^+^CD25^+^ mature Tregs from the indicated hBcl-2^Tg^ strains (n = 17, 11 and 6 for TGFβR1^WT^, TGFβR1^cKO^ and IL-2^KO^TGFβR1^cKO^), assayed in 6 independent experiments. **e**, Foxp3^-^CD25^+^ thymocytes from hBcl-2^Tg^ mice were injected into the thymus of B6 (n = 7) or γc^cKO^TGFβR1^cKO^ (n = 4) congenic hosts. Depicted are frequencies of Foxp3^+^CD25^+^ thymocytes developed from donor Foxp3^-^CD25^+^ thymocytes from 3 independent experiments. **f**, IL-2 protein in the serum. N = 3 per strain, representative of 2 independent experiments. **g**, Foxp3^-^CD25^+^ thymocytes from hBcl-2^Tg^ mice were injected into the thymus of B6 (n = 7) or CD25^KO^ (n = 6) congenic hosts. Donor origin cells were analyzed. Dots represent individual host mice from 3 independent experiments. **h**, Foxp3^-^CD25^+^ thymocytes from hBcl-2^Tg^Foxo^cDKO^ mice were injected into the thymus of B6 (n = 4) or CD25^KO^ congenic hosts that had received either Rat IgG (n = 3) or α-IL-2 (n = 3) antibodies. Frequency of donor origin Foxp3^+^CD25^+^ mature Tregs determined. Dots represent individual host mice from two experiments. **i**, CD69 expression on donor cells before and after intra-thymic injection. **j**, Schematic of two thymic Treg differentiation pathways. The primary developmental pathway is Foxo/TGFβ dependent, whereas the alternative developmental pathway is Foxo/TGFβ independent and requires excess IL-2. Two-tailed unpaired t-test, mean ± SEM (**a**, **b**, **c**, **d**, **e**, **f, g** and **h**).

We then examined whether TGF-β is required to disrupt agonist signaling and to induce in vivo generation of Foxp3^+^ cells (Fig. 5b and Extended Data Fig. 5a). For these experiments, we utilized TGF-β signaling-deficient thymocytes from 4-week-old TGFβR1^cKO^ mice before the onset of disease. In fact, TGF-β signaling deficiency both inhibited in vivo generation of Foxp3^+^ preT_reg_ cells and inhibited other consequences of signaling disruption as TGF-β signaling-deficient CD25^+^ precursors failed to differentiate into Foxp3^+^ cells, failed to upregulate n-Foxo1 and failed to downregulate CD69 (Fig. 5b and Extended Data Fig. 5a). We then confirmed these findings with thymocytes from γc-deficient γc^cKO^TGFβR1^cKO^ mice that never develop lethal lymphoproliferative disease^47^. First, we verified that γc was not required for generation of Foxp3^+^CD25^−^ preT_reg_ cells (Fig. 5c and Extended Data Fig. 5b)^14^ and then confirmed in γc^cKO^TGFβR1^cKO^ thymi that TGF-β signaling deficiency prevents generation of Foxp3^+^CD25^−^ preT_reg_ cells (Fig. 5c bottom and Extended Data Fig. 5b). Thus in vivo generation of Foxp3^+^CD25^−^ preT_reg_ cells requires TGF-β signaling to disrupt agonist signaling. Note that differentiation of Foxp3^−^CD25^+^ precursors into Foxp3^+^CD25^−/lo^ preT_reg_ cells requires initiation of Foxp3 expression and downregulation of agonist-induced CD25 expression, both of which result from TGF-β-mediated disruption of in vivo agonist signaling.

### Foxp3 gene induction by non-physiological amounts of IL-2

Exogenous IL-2 is known to induce Foxp3 expression, whereas endogenous IL-2 is thought to have similar in vivo effects^13,14,42,43^. Consequently, we were surprised that Foxp3 gene expression was initiated in preT_reg_ cells by agonist signaling disruption rather than by IL-2. Nonetheless we wondered if in vivo IL-2 might be capable of initiating Foxp3 gene expression by an alternative developmental pathway that did not require agonist signaling disruption.

To assess this possibility, we analyzed TGFβR1^cKO^ thymocytes which have a TGF-β signaling deficiency that prevents disruption of agonist signaling. We found that TGFβR1^cKO^ thymocytes lacked Foxp3^+^CD25^−^ preT_reg_ cells but nevertheless contained Foxp3^+^CD25^+^ mature T_reg_ cells (Fig. 5d and Extended Data Fig. 5c). These T_reg_ cells did not express molecular indicators of signaling disruption as they displayed decreased n-Foxo1, decreased CCR7 and elevated CD69 (Extended Data Fig. 5c). Moreover, generation of these Foxp3^+^ cells required IL-2 as they did not arise in IL-2^KO^TGFβR1^cKO^ thymocytes (Fig. 5d and Extended Data Fig. 5d). Thus Foxp3^+^ T_reg_ cells in TGF-β signaling-deficient mice were induced by IL-2 without apparent disruption of agonist signaling.

Even though Foxp3^+^CD25^+^ mature T_reg_ cells arose in intact TGFβR1^cKO^ thymi (Fig. 5d), Foxp3^−^CD25^+^ precursors from TGFβR1^cKO^ thymi did not differentiate into Foxp3^+^ cells after transfer into WT B6 thymi (Fig. 5b). To understand this difference, we wondered whether TGFβR1^cKO^ thymi contained more IL-2 than B6 thymi because undisrupted agonist signaling in TGFβR1^cKO^ mice may have stimulated excessive in vivo IL-2 production. In fact, TGFβR1^cKO^ mice do produce excessive IL-2 compared to B6 mice (Fig. 5e). To determine if such excessive amounts of in vivo IL-2 were required for alternative T_reg_ generation, we transferred WT CD25^+^ precursors into host thymi containing either excessive or normal amounts of endogenous IL-2 (Fig. 5f and Extended Data Fig. 5e). Transfer into γc^cKO^TGFβR1^cKO^ host thymi containing excessive IL-2 induced T_reg_ differentiation via the alternative pathway (Fig. 5f), whereas transfer into B6 thymi with normal IL-2 induced T_reg_ differentiation via the primary (preT_reg_) pathway (Fig. 5f). Similarly, we found that CD25^KO^ host mice contained excessive IL-2 (Extended Data Fig. 5f) and also induced differentiation of WT CD25^+^ precursors into T_reg_ cells via the alternative pathway (Fig. 5g,h and Extended Data Fig. 5g). Thus CD25^+^ precursors differentiate into mature T_reg_ cells via the primary pathway, except when they are signaled by excessive IL-2 to differentiate via the alternative pathway.

To confirm this conclusion, we transferred WT CD25^+^ precursors into γc^cKO^TGFβR1^cKO^ host thymi along with anti-IL-2 antibody (Fig. 5i). Control antibody did not alter differentiation of CD25^+^ precursors into mature T_reg_ cells via the alternative pathway (Fig. 5i), but anti-IL-2 antibody re-directed CD25^+^ precursors to differentiate into preT_reg_ cells via the primary pathway (Fig. 5i). Thus, excessive IL-2 induces CD25^+^ precursors to express Foxp3 and differentiate into Foxp3^+^CD25^+^ mature T_reg_ cells via the alternative pathway, preempting their differentiation into Foxp3^+^CD25^−^ preT_reg_ cells via the primary pathway.

Finally, we assessed whether T_reg_ development via the alternative pathway was Foxo-dependent. Whereas Foxo-deficient CD25^+^ precursors did not become Foxp3^+^ cells in thymi with normal IL-2 (Figs. 4d and 5j), they became Foxp3^+^CD25^+^ mature T_reg_ cells in CD25^KO^ thymi with excessive IL-2 and did so via the alternative pathway (Fig. 5j and Extended Data Fig. 5h). Notably, Foxo-deficient CD25^+^ precursors, unlike WT CD25^+^ precursors, differentiated in CD25^KO^ host thymi into Foxp3^+^CD25^+^ mature T_reg_ cells without downregulation of CD69 (Extended Data Fig. 5i), revealing that T_reg_ generation via the alternative pathway requires excessive IL-2 but is Foxo-independent (schematized in Extended Data Fig. 5j). Note that differentiation of Foxp3^−^CD25^+^ precursors into Foxp3^+^CD25^+^ mature T_reg_ cells requires initiation of Foxp3 expression and increased CD25 expression, both of which are signaled by excessive IL-2.

**Fig. 6:**
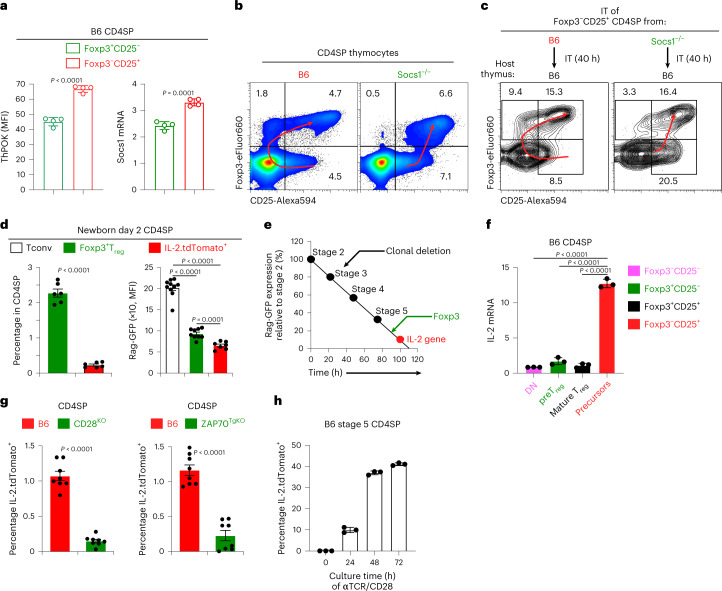
SOCS1 expression during primary T_reg_ differentiation and the development of IL-2^+^ T_eff_ cells in the thymus. **a**, ThPOK protein and SOCS1 mRNA expression in T_reg_ precursors and preT_reg_ cells of B6. CD4SP thymocytes. *n* = 4, representative of two independent experiments. **b**, Representative flow cytometry analysis of CD4SP thymocytes from the indicated strains. Red arrows indicate the primary (left) and alternative (right) developmental pathways. **c**, Purified Foxp3^−^CD25^+^ CD4 SP thymocytes from the indicated strain (CD45.2^+^) were injected into the thymus of CD45.2^+^ B6 congenic hosts. After 40 h, thymi were recovered and phenotype of donor populations was determined. Red arrows indicate the primary (left) and alternative (right) developmental pathways. Data are representative of two independent experiments with three host mice per group. **d**, Frequency of Foxp3^+^ T_reg_ cells and IL-2^+^ T_eff_ cells among CD4SP thymocytes from day 2 neonates (left) and their content of Rag-GFP (right). Dots represent individual mice from three independent experiments. **e**, Time (in hours) after *Rag2* gene cessation when Foxp3 and IL-2 gene expressions appear. Data are representative of three independent experiments. **f**, IL-2 mRNA in the indicated subsets of B6 CD4SP thymocytes (*n* = 3, representative of two independent experiments with four technical replicates). DN, double negative. **g**, IL-2^+^ T_eff_ cells among CD4SP thymocytes in B6, CD28^KO^ and ZAP70^TgKO^ mice. Dots represent individual mice from five independent experiments. **h**, Conventional IL-2^−^ CD4SP thymocytes from IL-2 reporter (IL-2.tdTomato) mice were stimulated in vitro with immobilized anti-TCR/CD28 monoclonal antibodies for the indicated time. Data show mean ± s.e.m. of triplicates cultures representative of four independent experiments. Numbers in the flow cytometry plots indicate percentages. Data were analyzed by two-tailed unpaired Student’s *t*-test, mean ± s.e.m. (**a**,**d**,**g**), one-way analysis of variance with Tukey’s post hoc test (**d**,**f**).

We then asked why only excessive IL-2 signaled CD25^+^ precursors to differentiate into mature T_reg_ cells via the alternative pathway. As a possible explanation, we considered that agonist-signaled CD25^+^ precursors might express high levels of the CD4 helper-lineage transcription factor, ThPOK, which is upregulated by TCR signaling^48–50^. Because ThPOK upregulates three members of the suppressor-of-cytokine signaling (SOCS) gene family which inhibit cytokine signal transduction^51^, increased SOCS expression can explain why alternative T_reg_ development pathway requires excessive IL-2. Indeed, ThPOK protein levels were significantly elevated in CD25^+^ precursors, as were SOCS1 mRNA levels (Fig. 6a).

**Extended Data Fig. 6:**
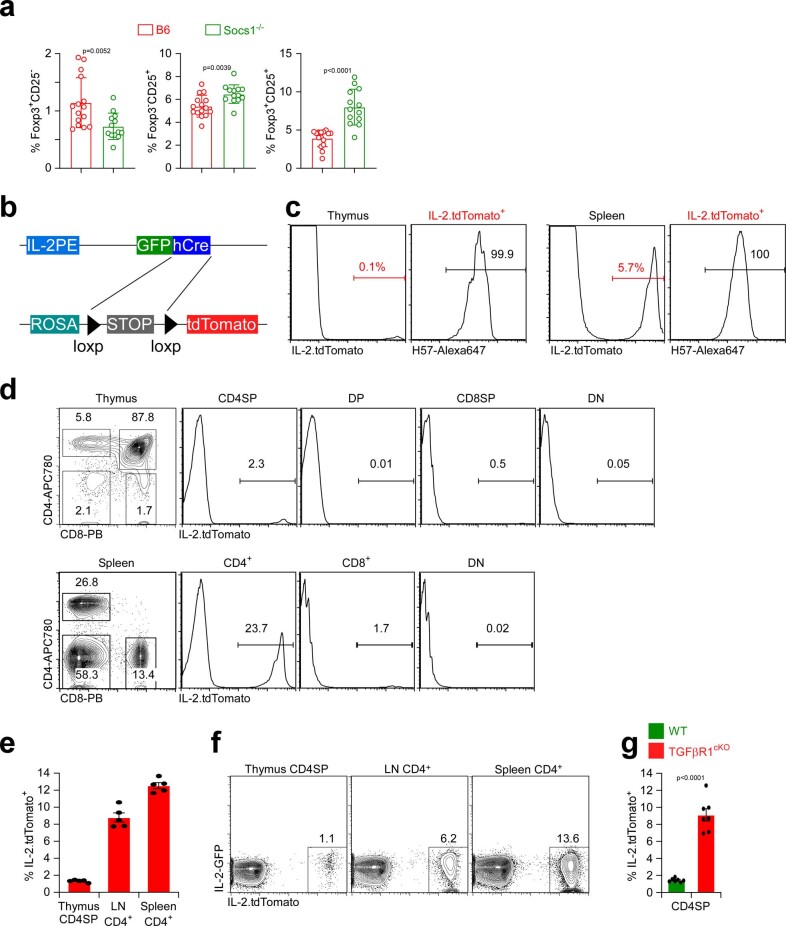
IL-2+ Teff cell generation. **a**, Characterization of Socs^-/-^ mice. Summary of flow cytometry analysis of CD4SP thymocytes from the indicated strains. Dots represent individual mice. **b**, A schematic representation of the IL-2 fate-mapping mouse line. Design of *IL2*-GFP-Cre BAC transgenic mice are provided in Experimental Procedures. IL-2PE, IL-2 promoter/enhancer. **c** and **d**, Flow cytometric analyses of thymocytes and spleen cells from IL-2 reporter (IL-2-tdTomato) mice. Representative of more than 3 independent experiments. **e** and **f**, IL-2^+^ cells among CD4^+^ T cells. **g**, Frequency of IL-2^+^ cells among CD4SP thymocytes from the indicated mice. Dots represent individual mice. Two-tailed unpaired t-test, mean ± SEM (**a** and **g**).

To determine whether elevated SOCS expression explained why CD25^+^ precursors required excessive IL-2 to induce Foxp3 expression, we examined thymic T_reg_ development in *Socs1*^−/−^ mice (Fig. 6b and Extended Data Fig. 6a). Notably, analysis of intact thymi indicated that B6 CD25^+^ precursors had developed into mature T_reg_ cells via the primary pathway, whereas SOCS1^−/−^CD25^+^ precursors developed into mature T_reg_ cells mainly via the alternative pathway (Fig. 6b). As confirmation, we intra-thymically transferred CD25^+^ precursors from SOCS1^−/−^ mice and found that, unlike B6 precursors, SOCS1^−/−^ precursors mostly differentiated into mature T_reg_ cells via the alternative pathway (Fig. 6c). Thus, elevated SOCS1 expression explains why CD25^+^ precursors are only signaled by excessive IL-2 to express Foxp3 and differentiate into T_reg_ cells via the alternative pathway.

In conclusion, initiation of Foxp3 gene expression under physiological conditions requires disruption of late agonist signaling by TGF-β to upregulate n-Foxo1 and to induce differentiation of CD25^+^ precursors into Foxp3^+^ preT_reg_ cells and then mature T_reg_ cells, which we refer to as the primary T_reg_ developmental pathway. However, under non-physiological conditions with excessive in vivo IL-2, Foxp3 gene expression is initiated independently of Foxo proteins and independently of signaling disruption to induce CD25^+^ precursors to differentiate directly into mature T_reg_ cells, which we refer to as the alternative T_reg_ developmental pathway.

### IL-2^+^ T_eff_ cells also require late agonist signaling

Having determined how autoreactive thymocytes are stimulated by late agonist signals to differentiate into Foxp3^+^ T_reg_ cells, we then assessed how autoreactive thymocytes are agonist-signaled to differentiate into T_eff_ cells that provide the IL-2 required by T_reg_ cells in the thymus^5,10^. To assess T_eff_ development in the thymus, we constructed IL-2 reporter mice with a modified *Rosa*-^loxp^STOP^loxp^-tdTomato locus and an *IL2*-GFP-Cre BAC transgene so that activation of the *Il2* gene would result in permanent labeling of IL-2-producing T_eff_ cells with tdTomato (Extended Data Fig. 6b). We refer to tdTomato^+^ cells as IL-2^+^ cells and found that all tdTomato^+^ cells were αβTCR^+^ cells (Extended Data Fig. 6c). In B6 mice, IL-2^+^ T_eff_ cells constituted 1–2% of CD4SP thymocytes and <15% of peripheral CD4^+^ T cells, which is consistent with recent findings^5^ (Extended Data Fig. 6d–f). As validation of IL-2 reporter mice, TGFβR1^cKO^ mice with excessive in vivo IL-2 also contained excessive IL-2^+^ thymocytes in 5–10-fold higher frequencies than B6 mice (Extended Data Fig. 6g).

To determine when IL-2^+^ T_eff_ cells arose, we compared Rag-GFP content in newly arising T_reg_ and T_eff_ cells in neonatal thymus that lacked recirculating cells. We found that IL-2^+^ T_eff_ cells were less frequent and contained significantly less Rag-GFP than T_reg_ cells (Fig. 6d). Based on Rag-GFP content, IL-2^+^ T_eff_ cells appeared significantly later than Foxp3^+^ T_reg_ cells, with Foxp3^+^ T_reg_ cells arising at 90 h and IL-2^+^ T_eff_ cells appearing at 100 h after Rag gene termination (Fig. 6e). Nevertheless, IL-2^+^ T_eff_ cells resembled Foxp3^+^ T_reg_ cells in initially arising from CD25^+^ precursors as revealed by IL-2 mRNA (Fig. 6f); in requiring in vivo CD28-dependent co-stimulation (Fig. 6g); and in requiring late agonist signaling (Fig. 6g). Because T_reg_ generation required disruption of agonist signaling, we wondered whether T_eff_ generation required persistent agonist signaling. We stimulated IL-2^−^ stage 5 thymocytes with immobilized anti-TCR/CD28 monoclonal antibodies and observed that persistent anti-TCR/CD28 stimulation did signal CD4 thymocytes to differentiate into IL-2^+^ T_eff_ cells, with more T_eff_ cells generated the longer anti-TCR/CD28 stimulation persisted (Fig. 6h). Thus, persistent agonist signaling induces T_eff_ cells.

**Fig. 7:**
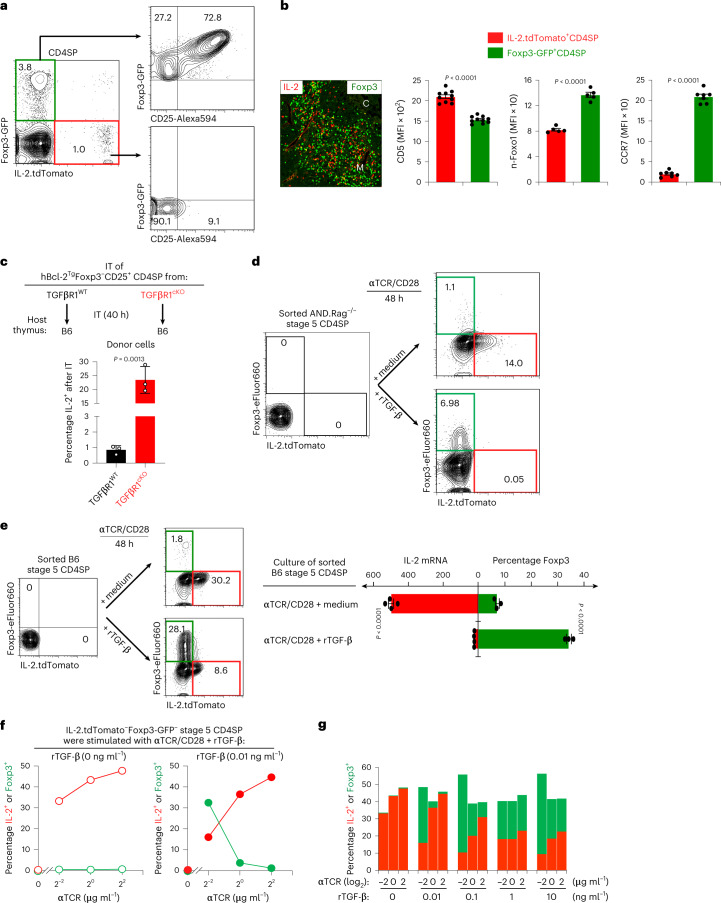
Signaling of T_reg_ versus T_eff_ cell generation. **a**, Flow cytometric analysis of CD4SP thymocytes from Foxp3-GFP/IL-2.tdTomato double-reporter mice. Representative of five independent experiments. **b**, Immunohistochemical assessment of thymic sections from Foxp3-GFP/IL-2.tdTomato double-reporter mice (left). Green identifies Foxp3-GFP^+^ thymocytes, red identifies IL-2.tdTomato^+^ thymocytes, C indicates the thymic cortex and M indicates the thymic medulla. Magnification ×10. Summary analysis of double-reporter thymocytes for expression of the indicated proteins (right). Dots represent individual mice from four independent experiments. **c**, Purified Foxp3^−^CD25^+^ CD4SP thymocytes from the indicated strain (CD45.2^+^) were injected into the thymus of CD45.1^+^ B6 congenic hosts (*n* = 3, for each donor cells, two independent experiments). After 40 h, thymi were recovered and IL-2 production from the donor populations was determined by intracellular staining. **d**,**e**, Purified conventional (Foxp3^−^IL-2^−^) stage 5 CD4SP thymocytes from indicated double-reporter (Foxp3-GFP/IL-2.tdTomato) mice were stimulated in vitro with immobilized anti-TCR/CD28 monoclonal antibodies alone or together with rTGF-β. Mean ± s.e.m., representative of three independent experiments. **f**,**g**, Purified conventional (Foxp3^−^IL-2^−^) B6 stage 5 CD4SP thymocytes from double-reporter (Foxp3-GFP/IL-2.tdTomato) mice were stimulated with different dose of anti-TCR alone or together with different dose of rTGF-β. Frequency of Foxp3^+^ T_reg_ cells and IL-2^+^ T_eff_ cells was analyzed after 48 h. Anti-CD28 was fixed at 25 μg ml^−1^. Data are representative of five independent experiments. Numbers in the flow cytometry plots indicate percentages. Data were analyzed by two-tailed unpaired Student’s *t*-test and show mean ± s.e.m. (**b**,**c**,**e**).

**Extended Data Fig. 7:**
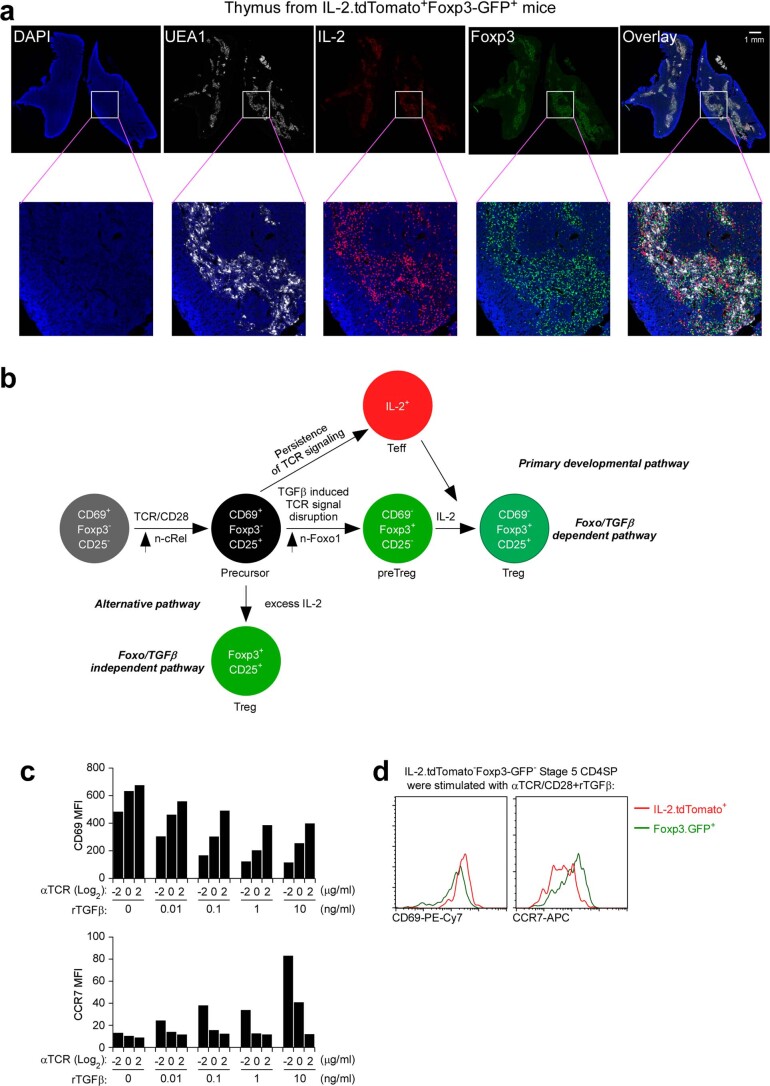
Differentiation of Foxp3+ Tregs and IL-2+ Teffs in the thymus. **a**, Immunohistochemical assessment of thymic sections from Foxp3-GFP/IL-2.tdTomato double-reporter mice (n = 3). UEA1, ulex europaeus agglutinin-1, labels medullar thymic epithelial cells (mTECs). Depicted representative of 3 independent experiments. **b**, Schematic of autoreactive thymocyte differentiation into Foxp3^+^ Tregs and IL-2^+^ Teffs. **c**, Purified conventional (Foxp3^-^IL-2^-^) stage 5 CD4SP thymocytes from double-reporter (Foxp3-GFP/IL-2.tdTomato) mice were stimulated with different doses of anti-TCR alone or together with different doses of rTGFβ. Surface CD69 and CCR7 were analyzed after 48 h. **d**, Surface CD69 and CCR7 expression on Foxp3^+^ Tregs and IL-2^+^ Teffs generated in **c** were analyzed. Representative of more than 5 independent experiments.

To directly compare T_reg_ and T_eff_ development, we generated Foxp3-GFP/IL-2.tdTomato double-reporter mice. In these double-reporter mice, GFP^+^ T_reg_ cells and tdTomato^+^ T_eff_ cells were distinct thymocyte subsets that were proximally localized to one another in the thymic medulla (Fig. 7a,b and Extended Data Fig. 7a). Notably, consistent with the T_eff_ requirement for persistent agonist signaling, IL-2^+^ T_eff_ cells expressed higher CD5, lower n-Foxo1 and lower CCR7 (Fig. 7b). Thus, T_reg_ and T_eff_ cells both require agonist signaling of late-stage thymocytes, but T_reg_ generation requires agonist signaling to be disrupted, whereas T_eff_ generation requires agonist signaling to persist without disruption.

We then assessed whether in vivo differentiation of agonist-signaled CD25^+^ precursors into IL-2^+^ T_eff_ cells is affected by TGF-β signaling (Fig. 7c). After intra-thymic transfer into B6 host thymi, TGFβR1^cKO^CD25^+^ precursors that fail to differentiate into Foxp3^+^ cells (Fig. 5b and Extended Data Fig. 5a) nonetheless become IL-2^+^ T_eff_ cells and do so in much higher frequencies than TGFβR1^WT^CD25^+^ precursors (Fig. 7c). Thus absent TGF-β signaling promotes in vivo differentiation of CD25^+^ precursors into IL-2^+^ T_eff_ cells (schematized in Extended Data Fig. 7b).

### Disruption versus persistence of agonist signaling

To further assess the impact of TGF-β on late agonist signaling, we in vitro stimulated both monoclonal and polyclonal Foxp3^−^IL-2^−^ stage 5 thymocytes in the presence and absence of rTGF-β (Fig. 7d,e). Anti-TCR/CD28 signaling alone induced IL-2^+^ T_eff_ cells containing high amounts of IL-2 messenger RNA (Fig. 7d,e); whereas anti-TCR/CD28 signaling plus rTGF-β induced Foxp3^+^ cells (Fig. 7d,e). Thus, the T_reg_/T_eff_ fate of late agonist-signaled thymocytes depends on whether or not TGF-β disrupts agonist signaling, for both polyclonal and monoclonal thymocytes expressing a single TCR specificity.

**Extended Data Fig. 8:**
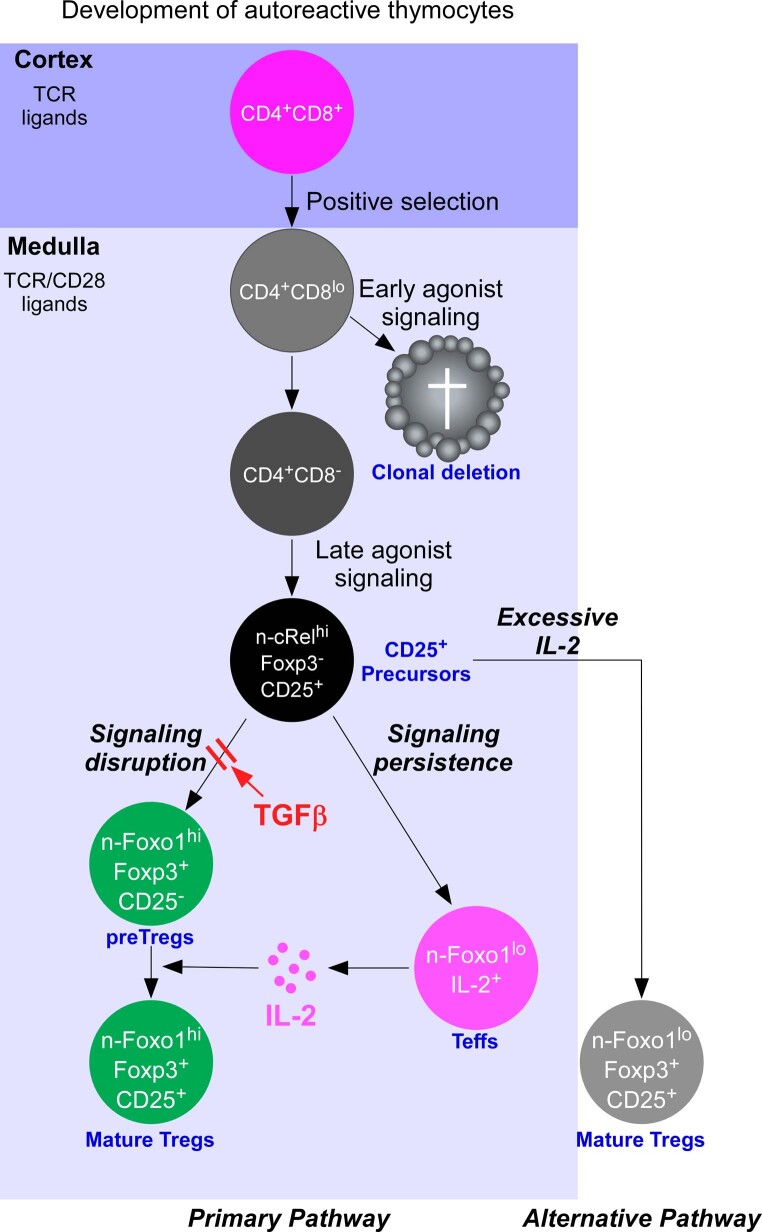
Schematic illustration of autoreactive thymocyte development in the normal thymus.

Finally, as TGF-β is present throughout the thymus^52^, IL-2^+^ T_eff_ cell generation would require late agonist signals that resisted signaling disruption by TGF-β, whereas Foxp3^+^ T_reg_ generation would require late agonist signals to be disrupted by TGF-β. To test this perspective, late-stage CD4^+^ thymocytes were stimulated with graded amounts of anti-TCR/CD28 monoclonal antibodies in the presence or absence of graded amount of rTGF-β (Fig. 7f,g). In the absence of rTGF-β, persistently signaled CD4^+^ thymocytes exclusively differentiated into IL-2^+^ T_eff_ cells (Fig. 7f); however, upon addition of TGF-β, CD4 thymocytes differentiated into both IL-2^+^ T_eff_ cells that were CD69^hi^CCR7^lo^ and into Foxp3^+^ T_reg_ cells that were CD69^lo^CCR7^hi^, with their relative frequencies dependent on TCR signaling intensity (Fig. 7f,g and Extended Data Fig. 7c,d). Thus, differentiation of agonist-signaled CD4^+^ thymocytes into IL-2^+^ T_eff_ cells or Foxp3^+^ T_reg_ cells depends on whether agonist signaling is strong enough to resist TGF-β-induced signaling disruption. Notably, the overall frequency of stimulated CD4SP thymocytes remained constant regardless of the fraction that became Foxp3^+^ T_reg_ cells or IL-2^+^ T_eff_ cells, suggesting that the same individual agonist-signaled CD4SP thymocytes can become either a T_reg_ or a T_eff_ cell (Fig. 7g). A schematic summary of this study is illustrated in Extended Data Fig. 8.

## Discussion

The present study documents that autoreactive CD4^+^ thymocytes achieve different developmental fates based on the timing and duration of agonist signaling in the thymus. Agonist signaling of thymocytes at early stages induce clonal deletion, whereas agonist signaling of thymocytes at late stages generates CD25^+^ precursors that differentiate into either Foxp3^+^ preT_reg_ cells or IL-2^+^ T_eff_ cells. Disruption of late agonist signaling in CD25^+^ precursors initiates Foxp3 gene expression and differentiation into Foxp3^+^ preT_reg_ cells then T_reg_ cells, whereas persistent late agonist signaling induces differentiation into IL-2^+^ T_eff_ cells. Notably, whether agonist signaling is disrupted or continues undisrupted depends on TGF-β which disrupts weaker agonist signaling but not stronger agonist signaling. As a result, TGF-β modulates in vivo T_reg_/T_eff_ lineage fate decisions and reveals that Foxp3^+^ preT_reg_ cells and IL-2^+^ T_eff_ cells are alternative lineage fates of agonist-signaled CD25^+^ precursors.

Because cRel and Foxo1 function together to initiate Foxp3 gene expression, we paid close attention to our observation that late agonist signaling upregulated n-cRel and that subsequent disruption of late agonist signaling was required to then upregulate n-Foxo1. As a result, initiation of Foxp3 gene expression required both agonist signaling and its subsequent disruption. Notably, we found that in vivo agonist signaling was disrupted by TGF-β, although the mechanism remains to be further clarified^45,46^. Of note TGF-β-induced disruption of agonist signaling explains how T_reg_ development by the primary pathway follows a curious ‘zigzag’ process^12^. That is, CD25 expression on thymocytes is upregulated by either TCR or IL-2 signaling, but TGF-β only inhibits TCR-induced CD25 and not IL-2-induced CD25 expression. Consequently, TGF-β-mediated disruption of agonist signaling in CD25^+^ precursors initiates Foxp3 gene expression and downregulates TCR-induced CD25 expression, causing thymocytes to become Foxp3^+^CD25^−^ preT_reg_ cells, which are then signaled by IL-2 to re-express CD25.

While our present study demonstrates that TGF-β signaling initiates Foxp3 gene expression by disrupting agonist signaling and upregulating n-Foxo1, we think that other mechanisms such as activation of SMAD molecules by TGF-β may be important for making the Foxp3 locus accessible to upregulated n-Foxo1 (ref. ^53^). In any event TGF-β-mediated upregulation of n-Foxo1 is a new mechanism of initiating Foxp3 expression and promoting T_reg_ differentiation in the thymus. Because generation of induced T_reg_ cells (iT_reg_ cells) also requires n-Foxo upregulation^34,35^, we think that TGF-β-mediated disruption of agonist signaling may also be necessary for induced T_reg_ development in the periphery.

Our present analysis of T_reg_ development contradicts the long-standing perspective that IL-2 signaling is the major initiator of Foxp3 gene expression. Our study also contradicts the perspective that thymic T_reg_ cells arise from two different pathways, with one pathway mediated by IL-2 signaled Foxp3^−^CD25^+^ precursors and the other pathway mediated by Foxp3^+^CD25^−^ preT_reg_ cells^13–15,42,43^. Instead, our present study documents that, under physiological conditions, there is only a single developmental pathway (referred to as the ‘primary’ pathway) in which agonist-signaled CD25^+^ precursors differentiate into Foxp3^+^CD25^−^ preT_reg_ cells without IL-2 when agonist signaling is disrupted, although IL-2 is subsequently required to signal preT_reg_ differentiation into Foxp3^+^CD25^+^ mature T_reg_ cells. Our present study documents that there also exists an IL-2-initiated alternative pathway, but this alternative pathway requires excessive non-physiological amounts of IL-2 that far exceed the amount of IL-2 in healthy mice and proceeds regardless of agonist signaling persistence or disruption. Notably, excessive IL-2 is required to initiate *Foxp3* gene expression when agonist signaling persists, because agonist signaling stimulates high expression of ThPOK-induced *SOCS* genes, such as *SOCS1*, which impair IL-2 signal transduction^54^. Consequently, the IL-2-initiated T_reg_ pathway is not physiologically relevant in normal circumstances.

It is generally thought that the different developmental fates of autoreactive thymocytes are correlated with their TCR affinities, as indicated by the affinity hierarchy: clonal deletion > T_eff_ cells > T_reg_ cells. However, the mechanism responsible for correlating TCR affinity with developmental fate is not known. Our present study provides a straightforward explanation for why different developmental fates are correlated with their TCR affinities. Thymocyte expression of TCR and CD4 steadily increase during thymic selection^55^ so that only the highest affinity autoreactive TCRs generate agonist signaling early in thymic selection when TCR and CD4 levels are lower, whereas lower affinity autoreactive TCRs can generate agonist signaling later in thymic selection when TCR and CD4 levels are higher. Among late-signaling autoreactive TCRs, persistence of agonist signaling requires higher affinity TCR and disruption of agonist signaling requires lower affinity TCR. Consequently, highest affinity TCRs generate early agonist signals that induce clonal deletion; intermediate affinity TCRs generate persistent late agonist signals that induce IL-2^+^ T_eff_ cells; and lower affinity TCRs generate disrupted late agonist signals that induce Foxp3^+^ T_reg_ cells. Thus, our present study explains the correlation of TCR affinity with the developmental fates of autoreactive thymocytes.

The observed proximity of Foxp3^+^ T_reg_ cells and IL-2^+^ T_eff_ cells in the thymus imply that these two subsets functionally regulate each other’s development. Notably, the amount of IL-2 in healthy mice is limited by T_eff_ cell number so that many preT_reg_ cells fail to receive IL-2 signals and consequently die from Foxp3-induced apoptosis^14,15^. The number of mature T_reg_ cells that arise and survive is directly related to the number of IL-2^+^ T_eff_ cells that are generated^5,56^.

In conclusion, our present study indicates that Foxp3^+^ preT_reg_ cells and IL-2^+^ T_eff_ cells are alternative lineage fates determined by disruption versus persistence of agonist signaling in CD25^+^ thymocyte precursors. Notably, a similar mechanism of signaling disruption versus persistence provides the basis for CD4/CD8 lineage fate determination during positive selection in the thymus, as detailed by the kinetic signaling model^1,57–60^. Thus, we consider signaling disruption versus persistence to be a general mechanism of lineage fate determination in the thymus.

## Methods

### Animals

C57BL/6 (B6) mice were obtained from the Frederick National Laboratory for Cancer Research; CBA/J, *Rag2*^−/−^, OT-II TCR^61^, Rip-mOVA^62^, *ZAP70*^−/−^, Nur77-GFP, *Il2rg*^−/−^, *Foxp3*^RFP^, *Rosa*^tdTomato^ (loxp-STOP-loxp), *Foxo1*^fl/fl^, *Foxo3*^fl/fl^, *Tgfbr1*^fl/fl^, *Il2*^−/−^ and *Cd25*^−/−^ mice were purchased from The Jackson Laboratory; Rag-GFP mice were provided by M. Nussenzweig^63^, *Tgfbr1*^fl/fl^ were initially provided by W. J. Chen, *Foxp3*^GFP^ mice were provided by V. K. Kuchroo^64^ and *Socs1*^*+/−*^*Ifng*^−/−^ mice were provided by J. Ihle^54^. E8III-Cre^58^, hBcl-2^Tg^, AND TCR transgenic^65^, PCC transgenic^66^, *B7*^DKO^, *Cd28*^−/−^, *Il2rg*^fl/fl^ (ref. ^67^) and *Socs1*^−/−^*Ifng*^−/−^ mice were bred in our own animal colony. All mice strains and breeding strategies are available in the Reporting Summary. All mice were analyzed without randomization or blinding. Most mice analyzed were 6–10 weeks of age and both sexes were used, except *Il2*^−/−^, *Cd25*^−/−^ and TGFβR1^cKO^ mice were analyzed around 3–4 weeks before onset of the disease. All animal experiments were approved by the National Cancer Institute Animal Care and Use Committee and mice were cared for in accordance with National Institutes of Health guidelines.

### Generation of E8_III_-ZAP70^Tg^ and IL-2 fate-mapping mice

Complementary DNA encoding mouse ZAP70 was introduced into the *Cd8*-E8_III_ promoter/enhancer construct TG-31 (ref. ^32^) to generate the E8_III_-ZAP70^Tg^. E8_III_-ZAP70^Tg^ was bred into *ZAP70*^−/−^ mice to generate ZAP70^TgKO^, so that only transgenic ZAP70 protein were expressed under the control of *Cd8*-E8_III_ promoter/enhancer.

For IL-2 fate-mapping mice, GFP-hCre cDNA (originally provided by N. Killeen) was inserted immediately downstream of the *Il2* ATG translational start site in a 215-kb B6 BAC (clone RP23-243E17) carrying the intact IL-2 gene. We used a codon-optimized ‘humanized’ Cre (hCre) to improve translational efficiency in eukaryotic cells. The modified BAC was then used to generate IL-2-Cre mice that were bred into *Rosa*^tdTomato^ (loxp-STOP-loxp) mice to generate IL-2 fate-mapping mice (IL-2-Cre*Rosa*^tdTomato^).

### Radiation bone-marrow chimeras

To assess TCR-Vβ-specific clonal deletion in ZAP70^TgKO^ thymocytes, lethally irradiated B6xCBA/J F1 (CD45.1^+^) recipient mice were reconstituted with a 2:1 mixture of T cell-depleted bone-marrow suspensions from B6xCBA/J F1 (CD45.1^+^) and ZAP70^TgKO^.B6 (CD45.2^+^) mice; 8 weeks after reconstitution, thymocytes of ZAP70^TgKO^.B6 (CD45.2^+^) origin were analyzed.

### Flow cytometry

Single-cell suspensions were prepared and stained with fluorochrome-conjugated antibodies with the following specificities (detailed information is provided in the section Reporting Summary): CD4 (GK1.5), CD8α (53-6-7), CD69 (H1.2F3), CCR7 (4B12), CD5 (53-7.3), TCRβ (H57-597), CD45.1 (A20), CD45.2 (104), Vα11 (RR8-1), Vβ3 (KJ25), Vβ5 (MR9-4), Vβ6 (RR4-7), Vβ8 (F23.1), Vβ11 (RR3-15), CD25 (PC61 & 7D4), Foxp3 (FJK-16s), ZAP-70 (1E7.2), Bim, mBcl-2 (100), Foxo1 (C29H4), cRel (REA397), ThPOK (T43-94) and IL-2 (JES6-5H4).

For cell surface staining of fresh cells, 1–2 × 10^6^ cells were incubated with 2.4G2 (anti-mouse F_c_γ III/II receptor) and stained with fluorochrome-conjugated antibodies. For staining with anti-CCR7(4B12), incubation was performed in the presence of 2.4G2 for 40 min at 37 °C. Dead cells were excluded by forward light-scatter gating and propidium iodide staining.

To detect transcription factors n-cRel, n-Foxo1 and Foxp3 and to detect intracellular membrane proteins Bim and Bcl-2, fresh cells were first surface stained in the presence of Ghost Dye Violet 510 and then fixed and permeabilized with the eBioscience Foxp3/Transcription Factor Staining Buffer Set according to the manufacturer’s instructions. Dead cells were excluded by forward light-scatter and Ghost Dye Violet 510 uptake, respectively.

Stained samples were analyzed on a LSRII or a LSRFortessa (BD Biosciences). Data were analyzed using FlowJo v.10.6.2 software.

### Timing of thymic selection

To determine the time for positive selection, CD69^+^CCR7^−^CD4^+^ thymocytes (stage 2) that represent the TCR-signaled thymocytes were considered as the starting point. Rag-GFP expression in stages 3, 4 and 5 relative to stage 2 CD4^+^ thymocytes was determined and those values were used for calculation of the timing with the formula: time = (100 – relative GFP expression) / 0.9.

To determine the time for clonal deletion and T_reg_ selection, Rag-GFP content in each stage Vβ5^+^ conventional thymocytes and Vβ5^+^Foxp3^+^ T_reg_ cells from Rag-GFP/Foxp3-RFP mice were measured and the values were used to calculate the timing as above.

To determine the timing for Foxp3^+^ T_reg_ cells and IL-2.tdTomato^+^ T_eff_ cell generation from the same thymus, thymocytes from day 2 newborn IL-2-Cre*Rosa*^dTomato^Rag-GFP mice were first surface stained and then fixed with 4% PFA at 20 °C for 10 min. PFA fixation retains intracellular GFP and tdTomato proteins. Fixed cells were then fixed/permeabilized with the eBioscience Foxp3/Transcription Factor Staining Buffer Set to stain nucleus Foxp3. Rag-GFP content in conventional CD4SP, Foxp3^+^ T_reg_ cells and IL-2^+^ T_eff_ cells was measured.

### Electronic cell sorting

To obtain CD4^+^ thymocytes from each developmental stage, total thymocytes from B6 mice were stained for CD4, CD8, CD69 and CCR7 and gated on CD4^+^ cells to electronically sort CD69^−^CCR7^−^ (stage 1), CD69^+^CCR7^−^ (stage 2), CD69^+^CCR7^lo^ (stage 3), CD69^+^CCR7^+^ (stage 4) and CD69^−/lo^CCR7^+^ (stage 5) thymocytes.

To purify precursors, total thymocytes from B6*Foxp3*^GFP^ and hBcl-2^Tg^*Foxp3*^GFP^ mice were depleted of CD8^+^ cells with anti-CD8 microbeads on MACS columns (Miltenyi Biotec); then stained for CD4, CD8, CD69 and CD25. Where indicated, Foxp3^GFP^^−^CD4^+^CD25^+^ CD4SP were further gated and electronically sorted to obtain purified CD4^+^CD25^+^CD69^−^ CD4SP or CD4^+^CD25^+^CD69^+^ CD4SP. Purified precursors were cultured in medium or with rhIL-2 (200 U ml^−1^) in the presence of gentamicin overnight. Where indicated, the in vitro-generated Foxp3^+^CD25^−/lo^ preT_reg_ cells were further sorted and cultured in the presence of rhIL-2 (200 U ml^−1^) for another overnight.

To purify stage 5 IL-2^−^Foxp3^−^ conventional T cells, whole thymocytes from IL-2-Cre*Rosa*^tdTomato^*Foxp3*^GFP^ mice were depleted of CD8^+^ cells; then stained for CD4, CD8 and CD69. IL-2.tdTomato^−^Foxp3^GFP−^CD69^−^CD4^+^CD8^−^ thymocytes were electronically sorted. All cells were electronically sorted on a FACSAria II.

### In vitro cultures

Different stage thymocytes were purified and stimulated with plate-bound anti-TCR (2 μg ml^−1^) + anti-CD28 (25 μg ml^−1^) or cultured in medium alone overnight in 1 ml cultures. To measure TCR-induced cell death, collected cells were stained with EtBr (1 μg ml^−1^). Relative frequency of EtBr^+^ cells was normalized as follows: (%EtBr^+^ (anti-TCR/CD28) − %EtBr^+^ (medium)) / (100 − %EtBr^+^ medium).

To induce Foxp3^+^ and IL-2^+^ T cells in vitro, purified stage 5 IL-2^−^Foxp3^−^ conventional T cells were stimulated with plate-bound anti-TCR (2 μg ml^−1^) + anti-CD28 (25 μg ml^−1^) in the absence or presence rTGF-β (10 ng ml^−1^) or anti-TGF-β (20 μg ml^−1^) plus SB431542 (5 μM) in 1-ml cultures for the indicated time. Where indicated, purified CD4SP was stimulated with graded amounts of anti-TCR plus a fixed dose of anti-CD28 (25 μg ml^−1^) in the presence of graded amounts of rTGF-β. For intracellular IL-2 staining, purified CD4SP thymocytes were stimulated with PMA (50 ng ml^−1^) + ionomycin (500 nM) for 3 h with BD GolgiStop Protein Transport Inhibitor presented for the last 2 h.

### Intra-thymic injections

Total thymocytes from Foxp3 reporter-positive B6, hBcl-2^Tg^, hBcl-2^Tg^Foxo^cDKO^ or hBcl-2^Tg^TGFβR1^cKO^ mice were depleted of CD8^+^ thymocytes to enrich CD4SP thymocytes. T_reg_ precursor cells were then sorted from purified CD4SP thymocytes and suspended in PBS. Thymic injections were performed as previously described^68^ into congenic B6, IL-2^KO^, γc^cKO^TGFβR1^cKO^ or CD25^KO^ hosts, 6–8 weeks of age. Then, 10 μl of donor cells suspended at 1 × 10^7^ ml^−1^ in PBS were injected into each thymic lobe using a Hamilton gastight syringe, 10-μl, cemented needle, 26s G, 2-inch beveled tip (Cole-Parmer). Where indicated, donor cells were co-injected with anti-IL-2 neutralizing antibodies (mixture of JES6-5H4, JESS6-1A12 and S4B6-1; 20 μg for each antibody per thymus) into the thymi of host mice, which also received a mixture of anti-IL-2-neutralizing antibodies (100 μg for each in 100 μl PBS per mouse) 1 h before intra-thymic injection followed by another dose 21 h later. Mice were euthanized after injection at the indicated time. CD4SP thymocytes from each injected host mouse were purified and stained with CD45.1 and CD45.2 to identify the donor and host cells.

### Confocal microscopy

To detect intracellular localization of transcription factors, purified Foxp3^GFP+^ thymocytes were fixed with 4% PFA at 20 °C for 30 min, permeabilizated with 0.5% Triton X-100 for 5 min at 20 °C, blocked with 3% goat serum (Jackson Immunoresearch) in PBS for 1 h at 20 °C, incubated with anti-Foxo1 (2880P, Cell Signaling) antibody for 1 h, washed with 0.1% Triton X-100 in PBS and then stained with an Alexa 488 (or Alexa 568)-conjugated goat anti-rabbit secondary antibody (Molecular Probes). Alternatively, cells were fixed with eBioscience Transcription Factor Fixation/Permeabilization buffer at 20 °C for 30 min, blocked with 3% goat serum (Jackson Immunoresearch) in eBioscience permeabilization buffer for 1 h at 20 °C, incubated with anti-Foxo1 antibody for 1 h, washed with eBioscience permeabilization buffer and then stained with an Alexa 488 (or Alexa 568)-conjugated goat anti-rabbit secondary antibody (Molecular Probes). Nucleus counterstaining was performed by incubating with 4,6-diamidino-2-phenylindole (DAPI) before mounting slides with Prolong Diamond Antifade Mountant (Thermo Fisher). Images were acquired using a Zeiss LSM 410 confocal microscope.

### Immunohistochemistry

Adult mice were transcardially perfused with PBS followed by 2% paraformaldehyde (PFA) in PBS under deep anesthesia with diethyl ether. The thymus was removed and fixed in 2% PFA in PBS for 2 h, immersed in sucrose gradient (10%, 20% and 30% wt/vol) in PBS sequentially 24 h for each step at 4 °C, then tissues were embedded in optimal cutting temperature compound (OCT). Frozen samples were sectioned at 6 μm. Cryosections were stained with Biotinylated UEA1 at 10 μg ml^−1^ (Vector Laboratories), chicken anti-GFP at 10 μg ml^−1^ (Abcam) and rabbit anti-RFP at 10 μg ml^−1^ (Rockland) followed by Alexa Fluor 647-conjugated streptavidin (Thermo Fisher), goat anti-chicken IgY Alexa 488 (Abcam) and goat anti-rabbit Alexa 546 (Thermo Fisher).

### Quantitative RT–PCR

Total RNA was isolated with an RNeasy Mini kit (QIAGEN) and treated with DNase I (Thermo Fisher) to eliminate possible genomic DNA contamination. cDNA synthesis was performed by superscript III First-Strand Synthesis System for RT–PCR kit with oligonucleotide dT primers (Invitrogen). TaqMan primers and probes (Applied Biosystems) were used for RT–PCR and samples were analyzed on a QuantStudio 6 Flex Real-Time PCR System (Applied Biosystems). The following TaqMan assays were used: *Il2* Mm00434256_m1 (cat. no. 4331182) and *Socs1* Mm00782550_s1 (cat. no. 4331182). All mRNA values were determined by quantitative PCR and are expressed relative to *Rpl13a* Mm01612986_gH (cat. no. 4331182).

### EdU labeling in vivo

To measure thymocyte proliferation, three mice per group received a single intraperitoneal injection of EdU (Thermo Fisher) at the dose of 50 mg kg^−1^ body weight. Thymocytes were collected 4 h or 15 h after injection and EdU staining was performed according to the manufacturer’s instructions (Thermo Fisher).

### ELISA

Thymus samples were lysed in lysis buffer containing 1% NP40, 10 mM Tris-HCL (pH 8.0), 150 mM NaCL, 10% glycerol, 5 mM EDTA plus proteinase inhibitor and protein concentration of the supernatant were determined. Quantification of IL-2 protein in thymic lysate and blood serum were determined using high sensitivity IL-2 mouse ELISA kit (Thermo Fisher).

### Quantification and statistical analysis

A Student’s *t*-test with unpaired two-tailed distributions or one-way analysis of variance with Tukey’s post hoc test were used for statistical analyses. *P* < 0.05 were considered significant. No statistical methods were used to predetermine sample sizes, but our sample sizes are similar to those reported in previous publications^47^.

### Reporting summary

Further information on research design is available in the Nature Portfolio Reporting Summary linked to this article.

## Online content

Any methods, additional references, Nature Portfolio reporting summaries, source data, extended data, supplementary information, acknowledgements, peer review information; details of author contributions and competing interests; and statements of data and code availability are available at 10.1038/s41590-023-01469-2.

## Supplementary information

Reporting Summary

## Data Availability

Mouse strains generated within this work are available for non-commercial research purposes following a reasonable request. Source data are provided with this paper.

## Source data

Source Data Fig. 1 Statistical source data.
Source Data Fig. 2 Statistical source data.
Source Data Fig. 4 Statistical source data.
Source Data Extended Data Fig. 1 Statistical source data.
Source Data Extended Data Fig. 2 Unprocessed images.
