# Supplementary material for: How autoreactive thymocytes differentiate into regulatory versus effector CD4+ T cells after avoiding clonal deletion
Source: Nat Immunol. 2023 Mar 23;24(4):637–51. doi: 10.1038/s41590-023-01469-2 (PMC10063450; doi:10.1038/s41590-023-01469-2)
Supplement: Supplementary file 1 — Reporting Summary [file 41590_2023_1469_MOESM1_ESM.pdf]

nature portfolio

Corresponding author(s): Alfred Singer

Last updated by author(s): Feb 10, 2023

Reporting Summary

Nature Portfolio wishes to improve the reproducibility of the work that we publish. This form provides structure for consistency and transparency in reporting. For further information on Nature Portfolio policies, see our Editorial Policies and the Editorial Policy Checklist.

Please do not complete any field with "not applicable" or n/a. Refer to the help text for what text to use if an item is not relevant to your study.
For final submission: please carefully check your responses for accuracy; you will not be able to make changes later.

Statistics

For all statistical analyses, confirm that the following items are present in the figure legend, table legend, main text, or Methods section.

n/a	Confirmed	
☐	☒	The exact sample size (n) for each experimental group/condition, given as a discrete number and unit of measurement
☐	☒	A statement on whether measurements were taken from distinct samples or whether the same sample was measured repeatedly
☐	☒	The statistical test(s) used AND whether they are one- or two-sided<br>*Only common tests should be described solely by name; describe more complex techniques in the Methods section.*
☒	☐	A description of all covariates tested
☐	☒	A description of any assumptions or corrections, such as tests of normality and adjustment for multiple comparisons
☐	☒	A full description of the statistical parameters including central tendency (e.g. means) or other basic estimates (e.g. regression coefficient) AND variation (e.g. standard deviation) or associated estimates of uncertainty (e.g. confidence intervals)
☐	☒	For null hypothesis testing, the test statistic (e.g. F, t, r) with confidence intervals, effect sizes, degrees of freedom and P value noted<br>*Give P values as exact values whenever suitable.*
☒	☐	For Bayesian analysis, information on the choice of priors and Markov chain Monte Carlo settings
☒	☐	For hierarchical and complex designs, identification of the appropriate level for tests and full reporting of outcomes
☒	☐	Estimates of effect sizes (e.g. Cohen's d, Pearson's r), indicating how they were calculated

Our web collection on statistics for biologists contains articles on many of the points above.

Software and code

Policy information about availability of computer code

Data collection	LSRII, LSRFortessa, FACSAria Fusion(Becton Dickinson), Zeiss LSM 410 confocal microscope, Zeiss LSM880 confocal microscopy, Zeiss AxioOberver Z1 equipped with Axiocam MRc5 camera , QuantStudio 6 Flex Real-time PCR System (Applied Biosystem) and SpectraMax iD3.
Data analysis	FlowJo 10.6.0, Prism 8 (Graph pad software), Adobe Photoshop, Adobe Illustrator, Canvas 12 and Adobe Illustrator

For manuscripts utilizing custom algorithms or software that are central to the research but not yet described in published literature, software must be made available to editors and reviewers. We strongly encourage code deposition in a community repository (e.g. GitHub). See the Nature Portfolio guidelines for submitting code & software for further information.

Data

Policy information about availability of data

All manuscripts must include a data availability statement. This statement should provide the following information, where applicable:
- Accession codes, unique identifiers, or web links for publicly available datasets
- A description of any restrictions on data availability
- For clinical datasets or third party data, please ensure that the statement adheres to our policy

Data sharing not applicable to this article as no datasets were generated or analysed during the current study.

Human research participants

Policy information about studies involving human research participants and Sex and Gender in Research.

Reporting on sex and gender	*Use the terms sex (biological attribute) and gender (shaped by social and cultural circumstances) carefully in order to avoid confusing both terms. Indicate if findings apply to only one sex or gender; describe whether sex and gender were considered in study design whether sex and/or gender was determined based on self-reporting or assigned and methods used. Provide in the source data disaggregated sex and gender data where this information has been collected, and consent has been obtained for sharing of individual-level data; provide overall numbers in this Reporting Summary. Please state if this information has not been collected. Report sex- and gender-based analyses where performed, justify reasons for lack of sex- and gender-based analysis.*
Population characteristics	*Describe the covariate-relevant population characteristics of the human research participants (e.g. age, genotypic information, past and current diagnosis and treatment categories). If you filled out the behavioural & social sciences study design questions and have nothing to add here, write "See above."*
Recruitment	*Describe how participants were recruited. Outline any potential self-selection bias or other biases that may be present and how these are likely to impact results.*
Ethics oversight	*Identify the organization(s) that approved the study protocol.*

Note that full information on the approval of the study protocol must also be provided in the manuscript.

Field-specific reporting

Please select the one below that is the best fit for your research. If you are not sure, read the appropriate sections before making your selection.

☒ Life sciences ☐ Behavioural & social sciences ☐ Ecological, evolutionary & environmental sciences

For a reference copy of the document with all sections, see nature.com/documents/nr-reporting-summary-flat.pdf

Life sciences study design

All studies must disclose on these points even when the disclosure is negative.

Sample size	The sample size for each experiment is indicated in the figure legends. The minimum sample size was chosen to reach statistical significance compared to control mice.
Data exclusions	No data were excluded
Replication	For all experiments, at least three replicates were analyzed in at least two independent experiments. The experimental findings were reliably reproduced.
Randomization	Animals were allocated to groups based on genotype.
Blinding	No blinding was used as no subjective scoring methods were used.

Reporting for specific materials, systems and methods

We require information from authors about some types of materials, experimental systems and methods used in many studies. Here, indicate whether each material, system or method listed is relevant to your study. If you are not sure if a list item applies to your research, read the appropriate section before selecting a response.

Materials & experimental systems

n/a	Involved in the study	
☐	☒	Antibodies
☒	☐	Eukaryotic cell lines
☒	☐	Palaeontology and archaeology
☐	☒	Animals and other organisms
☒	☐	Clinical data
☒	☐	Dual use research of concern

Methods

n/a	Involved in the study	
☒	☐	ChIP-seq
☐	☒	Flow cytometry
☒	☐	MRI-based neuroimaging

Antibodies

Antibodies used

anti-CD45.1 FITC (clone A20), BD Bioscience, Cat#553775, RRID: AB-395043
anti-CD45.1 Biotin (clone A20), BD Bioscience, Cat#553774, RRID: AB-395042
anti-CD45.1 PE/Cy7 (clone A20) BioLegend Cat# 110730, RRID: AB-1134168
anti-CD45.2 Alex594 (clone 104) BioLegend Cat#109850, RRID: AB_2629589
anti-CD45.2 PE (clone 104) BioLegend Cat#109808, RRID: AB_313445
anti-CD45.2 FITC (clone 104) BD Bioscience Cat#553772, RRID: AB_395041
anti-TCRab BV510 (clone H57-597) BD Bioscience Cat#563221, RRID: AB_2738078
anti-TCRab Alex594 (clone H57-597) BioLegend Cat#109238, RRID: AB_2563324
anti-TCRab eFluor 450 (clone H57-597) eBioscience Cat#48-5961-82, RRID: AB_11039532
anti-TCRab PE Cy/7 (clone H57-597) BD Bioscience Cat#560729, RRID: AB_1937310
anti-TCRab NA/LE (clone H57-597) BD Bioscience Cat#553166, RRID: AB_394678
anti-CD28 NA/LE (clone 37.51) BD Bioscience Cat#553294, RRID: AB_394763
anti-CD4 BV786 (clone GK1.5) BD Bioscience Cat#563331, RRID: AB_2738140
anti-CD4 APC-eFluor780 (clone GK1.5) eBioscience Cat#47-0042-82, RRID: AB_1272183
anti-CD5 APC (53-7.3) eBioscience Cat#17-0051-82, RRID: AB_469331
anti-CD8a Pacific Blue (clone 53-6.7) BD Bioscience Cat#558106, RRID: AB_397029
anti-CCR7 APC (clone 4B12) eBioscience Cat#17-1971-82, RRID: AB_469444
anti-CCR7 Biotin (clone 4B12) eBioscience Cat#13-1971-82, RRID: AB_466642
anti-CD69 PE (clone H1.2F3) BioLegend Cat#104508, RRID: AB_313111
anti-CD69 Pacific Blue (clone H1.2F3) BioLegend Cat#104524, RRID: AB_2074979
anti-CD69 PE/Cy7 (clone H1.2F3) BioLegend Cat#104512, RRID: AB_493564
anti-CD69 APC (clone H1.2F3) BioLegend Cat#104514, RRID: AB_492843
anti-Vα 11.1, 11.2 Biotin (clone RR8-1) BD Bioscience Cat#553221, RRID: AB_394716
anti-Vα 11.1, 11.2 FITC (clone RR8-1) BD Bioscience Cat#553222, RRID: AB_394717
anti-Vb3 PE (clone KJ25) BD Bioscience Cat#553209, RRID: AB_394709
anti-Vb5 FITC (clone MR9-4) BD Bioscience Cat#553189, RRID: AB_394697
anti-Vb5 Biotin (clone MR9-4) BD Bioscience Cat#553188, RRID: AB_394696
anti-Vb5 PE (clone MR9-4) BD Bioscience Cat#553190, RRID: AB_394698
anti-Vb6 Biotin (clone RR4-7) BD Bioscience Cat#553192, RRID: AB_394699
anti-Vb6 FITC (clone RR4-7) BD Bioscience Cat#553193, RRID: AB_394700
anti-Vb6 PE (clone RR4-7) BD Bioscience Cat#553194, RRID: AB_394701
anti-Vb8 PE (clone F23.1) BD Bioscience Cat#553862, RRID: AB_395098
anti-Vb11 PE (clone RR3-15) BD Bioscience Cat#553198, RRID: AB_394704
anti-CD25 Alex 594(clone PC61) BioLegend Cat#102045, RRID: AB_2563963
anti-CD25 BV510 (clone PC61) BD Bioscience Cat#563037, RRID: AB_2737969
anti-CD25 PE (clone PC61) BD Bioscience Cat#553866, RRID: AB_395101
anti-CD25 Biotin (clone 7D4) BD Bioscience Cat#553070, RRID: AB_394602
anti-CD25 PE/Cy7 (clone PC61) eBioscience Cat#25-0251-82, RRID: AB_469608
anti-CD132 PE (clone 4G3) BD Bioscience Cat#554457, RRID: AB_395404
anti-Foxp3 PE (clone FJK-16s) eBioscience Cat#12-5773-82, RRID: AB_467576
anti-Foxp3 eFluo660 (clone FJK-16s) eBioscience Cat#50-5773-82, RRID: AB_11218868
anti-ZAP-70 PE (clone 1E7.2) BioLegend Cat#313404, RRID: AB_2304732
anti-ThPok Alex 647 (T43-94) BD Bioscience Cat#565500), RRID: AB_2739268
anti-Bim BD Bioscience Cat#559685, RRID: AB_397305
anti-Bcl-2 PE (clone 100) BioLegend Cat#658707, RRID: AB_2563281
anti-Foxo1 (clone C29H4) Cell signaling Cat#2880P, RRID: AB_2106495
anti-cRel PE (clone REA397) Miltenyi Biotec Cat#130-124-715, RRID: AB_2651454
Normal Goat Serum Jackson ImmunoResearch Cat#005-000-121, RRID: AB_2336990
anti-GFP abcam Cat#ab13970, RRID: AB_300798
Goat anti-chicken IgY Alex 488 abcam Cat#ab150173, RRID: AB_2827653
anti-RFP ROCKLAND Cat#600-401-379, RRID: AB_2209751
Goat anti-rabbit IgG(H+L) Alex 488 Thermo Fisher Cat#A-11070, RRID: AB_142134
Goat anti-rabbit IgG(H+L) Alex 568 Thermo Fisher Cat#A-11011, RRID: AB_143157
Goat anti-rabbit IgG(H+L) Alex 546 Thermo Fisher Cat#A-11071, RRID: AB_2534115
anti-IL-2 Alex 647 (clone JES6-5H4) BioLegend Cat#503814, RRID: AB_493517
anti-TGFb (clone 1D11) Thermo Fisher Cat#MA5-23795, RRID: AB_2609812
anti-IL-2 (clone JES6-5H4) BioXcell Cat#BE0042, RRID: AB_1107703
anti-IL-2 (clone JES6-1A12) BioXcell Cat#BE0043, RRID: AB_1107702
anti-IL-2 (clone S4B6-1) BioXcell Cat#BE0043-1, RRID: AB_1107705

Validation

All antibodies are commercially available and have been validated by the manufacture or in previous reports.

Animals and other research organisms

Policy information about studies involving animals; ARRIVE guidelines recommended for reporting animal research, and Sex and Gender in Research

Laboratory animals

Mouse: CD45.1+/B6, Charles River #564
Mouse: CD45.2+/B6, Charles River #027

Mouse: CBA/J, Jackson Laboratory #000656
Mouse: B6/CBA/J, this paper
Mouse: Rag-GFP, Yu et al., 1999
Mouse: hBcl-2Tg, Sentman et al.,1991
Mouse: Rosa26tdTomato (Loxp-STOP-Loxp), Jackson Laboratory #007908
Mouse: Il2-/-, Jackson Laboratory #002229
Mouse: AND TCRTg, Kaye et al., 1989
Mouse: PCCTg, Oehen et al., 1996
Mouse: OTII TCRTg, Jackson Laboratory #004194
Mouse: RIP-mOVA, Jackson Laboratory #005431
Mouse: ZAP70-/-, Jackson Laboratory #004225
Mouse: ZAP70Tg, this paper
Mouse: ZAP70-/-ZAP70Tg (ZAP70TgKO), this paper
Mouse: ZAP70+/+ZAP70Tg (ZAP70TgWT), this paper
Mouse: E8III-Cre, Park et al., 2010
Mouse: Nur77-GFP, Jackson Laboratory #016617
Mouse: Il2rgfl/fl, McCaughtry et al., 2012
Mouse: Il2rgfl/flE8III-Cre (gccKO), this paper
Mouse: Il2rgfl/flhBcl-2TgE8III-Cre (gccKOhBcl-2Tg), this paper
Mouse: Tgfbr1fl/fl, Jackson Laboratory #028701
Mouse: Tgfbr1fl/flE8III-Cre (TGFbR1cKO), this paper
Mouse: Il2-/-Tgfbr1fl/flE8III-Cre (IL-2KOTGFbR1cKO), this paper
Mouse: Il2rgfl/flTgfbr1fl/flE8III-Cre (gccKOTGFbR1cKO), this paper
Mouse: hBcl-2TgTgfbr1fl/flE8III-Cre (hBcl-2TgTGFbR1cKO), this paper
Mouse: Il2rgfl/flhBcl-2TgTgfbr1fl/flE8III-Cre (gccKOhBcl-2TgTGFbR1cKO), this paper
Mouse: Rag-/-, Jackson Laboratory #008449
Mouse: Cd25-/-, Jackson Laboratory #002462
Mouse: Cd28-/-, Shahinian et al., 1993
Mouse: B7DKO, Borriello et al., 1997
Mouse: Socs1-/-Ifng-/-, Marine et al., 1999
Mouse: Foxo1fl/fl, Jackson Laboratory #024756
Mouse: Foxo3fl/fl, Jackson Laboratory #024668
Mouse: Foxo1fl/flFoxo3fl/flE8III-Cre (FoxocDKO), this paper
Mouse: Foxo1fl/flFoxo3fl/flhBcl-2TgE8III-Cre (hBcl-2TgFoxocDKO), this paper
Mouse: Foxp3-RFP backcrossed to B6, Jackson Laboratory #008374
Mouse: Foxp3-RFP backcrossed to Rag-GFP, this paper
Mouse: Foxp3-RFP backcrossed to Nur77-GFP, this paper
Mouse: Foxp3-GFP backcrossed to B6, Bettelli et al., 2006
Mouse: Foxp3-GFP backcrossed to B7DKO, this paper
Mouse: Foxp3-GFP backcrossed to hBcl-2Tg, this paper
Mouse: Foxp3-GFP backcrossed to FoxocDKO, this paper
Mouse: Foxp3-GFP backcrossed to hBcl-2TgFoxocDKO, this paper
Mouse: Foxp3-GFP backcrossed to TGFbR1cKO, this paper
Mouse: Foxp3-GFP backcrossed to hBcl-2TgTGFbR1cKO, this paper
Mouse: IL-2-Cre, this paper
Mouse: IL-2-CreRosatdTomato, This paper
Mouse: IL-2-CreRosatdTomato backcrossed to Rag-GFP, this paper Mouse: IL-2-CreRosatdTomato backcrossed to Foxp3-GFP, this paper Mouse: IL-2-CreRosatdTomato backcrossed to ZAP70TgKO, this paper Mouse: IL-2-CreRosatdTomato backcrossed to Cd28-/-, this paper Mouse: IL-2-CreRosatdTomato backcrossed to TGFbR1cKO, this paper Mouse: B6/CBA/J + ZAP70TgKO to B6/CBA/J F1 mixed chimera, this paper Mouse: AND.H-2b/kPCC-Rag+/+, this paper
Mouse: AND.H-2b/kPCC+Rag+/+, this paper
Mouse: OTII.H-2b/bOVA-Rag+/+, this paper
Mouse: OTII.H-2b/bOVA+Rag+/+, this paper

Wild animals	No wild animals were used in this study.
Reporting on sex	Both male and female mice were used and analyzed at age 6-10 weeks old. Animals that develop autoimmune disease were used at 3-4 weeks before the onset of disease.
Field-collected samples	No field collected samples were used in this study.
Ethics oversight	All animal experiments were approved by the National Cancer Institute Animal Care and Use Committee and were maintained in accordance with US National institutes of Health guidelines.

Note that full information on the approval of the study protocol must also be provided in the manuscript.

Flow Cytometry

Plots

Confirm that:

☒ The axis labels state the marker and fluorochrome used (e.g. CD4-FITC).

☒ The axis scales are clearly visible. Include numbers along axes only for bottom left plot of group (a 'group' is an analysis of identical markers).

☒ All plots are contour plots with outliers or pseudocolor plots.

☒ A numerical value for number of cells or percentage (with statistics) is provided.

Methodology

Sample preparation	Single cell suspension were prepared in cold HBSS supplemented with 0.5% BSA and 0.5% NaN3.
Instrument	LSRII, LSRFortessa, FACSAria Fusion(Becton Dickinson).
Software	FlowJo 10.6.0
Cell population abundance	>95% on sorted cells, which was determined by flow cytometry analysis on post sorted cells.
Gating strategy	Live cells were defined by FSC gating and staining with propidium iodide or LIVE/DEAD Fixable Aqua Dead Cell Stain Kit. All gating strategies are stated in the manuscript.

☒ Tick this box to confirm that a figure exemplifying the gating strategy is provided in the Supplementary Information.

This checklist template is licensed under a Creative Commons Attribution 4.0 International License, which permits use, sharing, adaptation, distribution and reproduction in any medium or format, as long as you give appropriate credit to the original author(s) and the source, provide a link to the Creative Commons license, and indicate if changes were made. The images or other third party material in this article are included in the article's Creative Commons license, unless indicated otherwise in a credit line to the material. If material is not included in the article's Creative Commons license and your intended use is not permitted by statutory regulation or exceeds the permitted use, you will need to obtain permission directly from the copyright holder. To view a copy of this license, visit http://creativecommons.org/licenses/by/4.0/
